# Quantitative genetic analysis of attractiveness of yeast products to *Drosophila*

**DOI:** 10.1093/genetics/iyae048

**Published:** 2024-04-01

**Authors:** Weiru Yan, Yishen Li, Edward J Louis, Charalambos P Kyriacou, Yue Hu, Rebecca L Cordell, Xiaodong Xie

**Affiliations:** Institute of Genetics, School of Basic Medical Sciences, Lanzhou University, Lanzhou 730000, China; Department of Genetics & Genome Biology, University of Leicester, Leicester LE1 7RH, UK; Department of Genetics & Genome Biology, University of Leicester, Leicester LE1 7RH, UK; Department of Genetics & Genome Biology, University of Leicester, Leicester LE1 7RH, UK; Department of Genetics & Genome Biology, University of Leicester, Leicester LE1 7RH, UK; Department of Genetics & Genome Biology, University of Leicester, Leicester LE1 7RH, UK; School of Chemistry, University of Leicester, University Road, Leicester LE1 7RH, UK; Institute of Genetics, School of Basic Medical Sciences, Lanzhou University, Lanzhou 730000, China

**Keywords:** *Drosophila melanogaster*, *Saccharomyces cerevisiae*, quantitative trait loci, olfactory-induced preference, reciprocal hemizygosity assay, fermentation

## Abstract

An attractive perfume is a complex mixture of compounds, some of which may be unpleasant on their own. This is also true for the volatile combinations from yeast fermentation products in vineyards and orchards when assessed by *Drosophila*. Here, we used crosses between a yeast strain with an attractive fermentation profile and another strain with a repulsive one and tested fly responses using a T-maze. QTL analysis reveals allelic variation in four yeast genes, namely *PTC6*, *SAT4*, *YFL040W*, and *ARI1*, that modulated expression levels of volatile compounds [assessed by gas chromatography–mass spectrometry (GC–MS)] and in different combinations, generated various levels of attractiveness. The parent strain that is more attractive to *Drosophila* has repulsive alleles at two of the loci, while the least attractive parent has attractive alleles. Behavioral assays using artificial mixtures mimicking the composition of odors from fermentation validated the results of GC–MS and QTL mapping, thereby directly connecting genetic variation in yeast to attractiveness in flies. This study can be used as a basis for dissecting the combination of olfactory receptors that mediate the attractiveness/repulsion of flies to yeast volatiles and may also serve as a model for testing the attractiveness of pest species such as *Drosophila suzukii* to their host fruit.

## Introduction

The attractiveness of fungal volatiles to insects has been extensively studied in recent years (Bergström 2008; Beck and Vannette 2017; Madden *et al.* 2018; Günther *et al*. 2019). Ecological studies have shown that *Saccharomyces* and *Drosophila* co-habit the same niches, such as vineyards and orchards (Goddard and Greig 2015; Lam and Howell 2015). It is commonly observed that flies hover around the rotten fruit with the larvae thriving within it. The key connection between the two organisms are the volatile compounds from fermentation (Christiaens *et al*. 2014). In an oxygen-rich environment, *Saccharomyces cerevisiae* ferments sugars via the Crabtree effect, which generates a large amount of ATP despite not being an efficient route for utilizing carbon resources (Pfeiffer *et al.* 2001). A series of volatile compounds are synthesized in this process. Yeasts utilize amino acids, sugars, and fatty acids to produce higher alcohols, esters, carbonyls, sulfur compounds, and ethanol (Swiegers and Pretorius 2005). As this process consumes ATP and uses carbon, whether the generation of volatile compounds is a side product of metabolism or whether it facilitates ecological and physiological roles remains unclear (Saerens *et al*. 2010).

Yeast is a key part of the *Drosophila* diet and benefits life span and adult body weight (Anagnostou *et al*. 2010). Yeast not only enhances *Drosophila* longevity but also increases survival rates of larvae in unfavorable environments (Rohlfs and Kurschner 2010). Previous reports suggested that yeast can detoxify mycotoxins, which may improve the viability of flies in their natural habitats (Hatout and Aly 2014). A common view is that the dispersal of yeasts relies on engagement with insects. It is thought that volatile compounds from fermentation can be considered as a type of chemical communication, facilitating mobility of yeast (Buser *et al*. 2014; Christiaens *et al*. 2014). Natural yeast mating has been reportedly found within insect intestines, which does not easily occur in other natural environments (Cubillos *et al*. 2009; Stefanini *et al*. 2012). However, the interaction between *Drosophila* and yeast cannot be treated as a typical mutualism or predator–prey interaction and needs further investigation.

There are several reports on the interactions between yeast and flies through volatiles. *Drosophila* can be attracted by acetates generated by yeasts (Christiaens *et al*. 2014) in both wild and laboratory environments (Buser *et al*. 2014). The levels of attraction of *Drosophila* vary with yeast species (Palanca *et al*. 2013), and *Drosophila* reproduction is enhanced by the presence of yeast (Buser *et al*. 2014). Furthermore, insects such as *Drosophila* may disperse yeasts either internally by ingestion or externally by adherence, providing a possible route for explaining the global distribution of the *Saccharomyces* genus (Reuter *et al*. 2007; Goddard *et al*. 2010). Decaying fruits harboring fungi act as sites for *Drosophila* courtship and reproduction, with both adults and larvae feeding on the host substrate and its associated microorganisms (Ganter 2006; Markow and O’Grady 2008). Fertilized female flies of fruit-associated species prefer oviposition sites colonized by fermenting yeast (Becher *et al*. 2012). One study reported that fermented-like odor can attract fruit flies without the presence of yeast (Stökl *et al*. 2010). Another mimicked the volatile composition of a rainforest orchid and studied its attractiveness to one fly species (Martos *et al*. 2015).

The interaction with yeasts is triggered by the fly's olfactory and gustatory responses (Masse *et al*. 2009). The olfactory receptors of *Drosophila* are sensitive to esters and higher alcohols produced by yeasts (Hallem *et al*. 2004). *Drosophila* are also resistant to alcohol because their endogenous alcohol dehydrogenase facilitates survival in high-ethanol environments (Devineni and Heberlein 2009; Joseph *et al*. 2009). Fertilized female flies are attracted to acetic acid via their gustatory system but are repelled when the signal is detected by olfactory receptors (Piškur *et al*. 2006; Joseph *et al*. 2009). Previous studies have listed a series of compounds that attract *Drosophila*, such as 2-phenylethanol, acetaldehyde, ethyl esters, ethyl acetate, isoamyl acetate, and volatile acids, most of which can be found in the odor of yeast fruit fermentation (Stensmyr *et al*. 2003; Lebreton *et al*. 2012).

To explore the potential determining factors of the interaction between *Drosophila* and yeast, we used a quantitative genetic approach to investigate the allelic variation in yeast strains that underlies the attraction of fruit flies to volatile compounds as measured via a T-maze. An artificial mixture mimicking the volatile compound concentration and composition of the parental lines was applied in the T-maze to verify the results of our gas chromatography–mass spectrometry (GC–MS) analyses. Our results suggest that *Drosophila* were able to distinguish the allelic variation underlying parental volatile compound.

## Materials and methods

### Yeast strains and culture

Five *S. cerevisiae* strains were selected from the Saccharomyces Genome Resequencing Project (Carter 2008): Wine European (WE; DBVPG6765), West Africa (WA; DBVPG6044), North America (NA; YPS128), SA (Y12; Sake), and YJM978 (clinical isolate from the USA). The laboratory strain, S288C, was also included in this study. The F1 progeny of WE and NA was used as the QTL mapping population, as described previously (Cubillos *et al*. 2011; Jara *et al*. 2014). All strains were maintained on YPD solid media (2% glucose, 2% peptone, 1% yeast extract, and 2% agar). For fermentation, 50 mL sterilized diluted concentrated grape juice (Wilko 0483794) was used. Sterilization was performed using 1 mL/L dimethyl dicarbonate (517127, Merck, UK), which is regularly used in wine making. Yeast strains were precultured in liquid YPD media 1 day before the fermentation; 2 × 10^4^ cells/mL were inoculated into grape juice from each strain and left for 7 days at 30°C in a shaking incubator (250 rpm) for fermentation.

### Fly maintenance

The strain M1201 was established from wild-captured flies in the summer of 2012 near Trento in the North of Italy. Several gravid females were used to found the line. Fly stocks were maintained at 25°C in the presence of 70% relative humidity under a 12-h light:12-h dark photoperiod on cornmeal food. Flies were collected on the day of eclosion via anesthetization with CO_2_ and transferred to new food vials. They were sorted based on sex after 2–4 days, transferred to a new clean vial, and starved for 24 h before the preference assay.

### T-maze preference assay

T-maze is a binary choosing apparatus allowing testing animal to make a decision depending on the stimulants on different sides and then recording the number of individuals to evaluate preference. An eight-channel T-maze was produced using 3D printing (3D Quick Printing, UK, Supplementary Fig. 1a). For the preference assay, each 15-mL centrifuge tube was loaded with 20 male or fertilized female flies (previously mated in the incubation tubes) without anesthetization and attached to the main column of the apparatus. The fermented grape juice was diluted 100 times, and 1.5 mL was deposited in the small container of each chamber in each arm of the maze. The T-maze was placed in a dark room for 6 h to allow flies to make choices. Then, the flies were introduced into the column and allowed to choose between two volatile stimuli. The number of flies in the arm of the maze chamber was counted, and the preference index (PI) was calculated using the following formula:


Preferenceindex(PI)=(PM−0.5)/[(PM+0.5)−(2PM×0.5)]

where PM is the proportion of flies in the right direction (arbitrarily chosen).

### Headspace-Solid Phase Microextraction-gas chromatography-mass spectrometry (Headspace-SPME-GC-MS)

After fermentation, 2 mL of the supernatant was collected via centrifugation (13,000 rpm, 2 min) and aspirated to a 10-mL sample vial (5188–5392, Agilent, UK). The SPME fibers used in this study were purchased from Supelco (57295-U, Merck). The StableFlex fibers were coated with an 85-µm film of carboxen/polydimethylsiloxane. All samples were equilibrated at 80°C for 10 min with agitation at 500 rpm using an autosampler prior to injection. The headspace was sampled for 60 min onto the SPME fiber. Gas chromatography–mass spectrometry analysis was performed using an Agilent 7890A GC and 5975C MS with a CTC-PAL autosampler (Agilent Technologies, UK). A DB-WAX Ultra Inert column (*l* = 30 m, I.D. = 0.25 mm, dF = 0.25 µm, Agilent Technologies, UK) was used for the analysis. The injector temperature was set at 260°C, while the oven was held at 30°C for 5 min, which was first increased at 4°C/min to 200°C and then at 15°C/min to 250°C, and finally held at 250°C for 5 min. Two internal standards, namely octanol-d17 (448222, Merck, UK) and acetophenone-d8 (sc-227203, Santa Cruz Biotechnology, TX, USA), were integrated into the samples to modulate the variations. Compounds were initially identified by comparing with the MS library [National Institute of Standards and Technology 11 (NIST11)] and then identified by testing commercial standards (Supplementary Table 1). The compounds were further quantified using calibration lines generated using a series of dilutions and processed using MassHunter Quantitative Analysis (Agilent, CA, USA).

### Bioinformatics of R/QTL

The R/QTL files were manually curated by matching all phenotypic values (PI and concentrations of quantified compounds) of segregants with their genotype data and fed into ShmooTL, which provides a recently developed R pipeline for QTL mapping analysis (Hu 2020). The full technical information regarding ShmooTL is available at GitHub (https://github.com/gact/shmootl). In total, 179 high-resolution melting curves of polymerase chain reaction (PCR) were used as markers, filtered from those previously identified among the founder strains (Cubillos *et al*. 2011). A 1.5-LOD interval drop was applied from peak marker with a 40 kb spanning window to locate the QTL intervals. The genes located in the selected QTL intervals were annotated, and Gene Ontology (GO) terms were text-mined from SGD database (https://www.yeastgenome.org/) with keywords (such as metabolism and glycolysis).

### Reciprocal hemizygosity assay

The reciprocal hemizygosity assay (RHA) was designed to identify allelic differences that are responsible for phenotypic variation. Briefly, haploid versions of the parental strains (WE, *hoΔ::HphMX MATa*, and NA, *hoΔ::HphMX MATalpha*) were used to delete each target gene and construct all possible combinations of single deletions. Primers for gene deletions using selectable markers were designed to enable site-specific recombination at target loci. Primers were located 200–300 bp upstream or downstream of the target gene (Supplementary Table 3). The genomic DNA of the corresponding deletion strain obtained from the Genome Deletion Collection was the template used for amplifying the KanMX cassette (Giaever and Nislow 2014). Yeast transformation was performed using PEG–LiAc and a single-stranded DNA carrier, as described elsewhere (Gietz and Schiestl 2007). The heat-shock conditions were optimized before transformation (WE: 42°C, 18 min; WA: 42°C, 20 min). The PCR product containing the target homology fragments at the ends is the target for transformation. The transformed cells were plated onto YPD-G418-HYG [YPD agar with 0.16 g/L G418 disulfate salt (A1720, Merck, UK) and 200 mg/L hygromycin B (10687010, ThermoFisher, UK)]. Diagnostic PCR was used to verify the gene knockout after transformation. Mutated parental strains were crossed to generate reciprocal hemizygous strains and selected on drug-containing plates (YPD-G418-HYG). The diploid hybrid strains were confirmed using Mating-type (MAT) locus PCR, and deletions of the target genes were confirmed using PCR with specific primer pairs (Supplementary Tables 3 and 6). The verified pairs of hemizygous strains were inoculated into the prepared grape juice for preference assays and GC–MS profiling.

### Statistical analysis

Statistical analysis of preference assays was performed using 16 replicates and a one-sample *t*-test (theoretical error = 0.058, measured by depositing grape juice on each side of the T-maze used in the PI analysis) in GraphPad Prism 9 for Windows. Principal component analysis (PCA) was performed using the XLSTAT for analysis of volatile compounds’ concentration and to discriminate strains. The concentration of each compound was visualized using Prism 9 and compared between strains using a *t*-test. Gas chromatography–mass spectrometry analysis was performed using MassHunter (Agilent, CA, USA).

## Results

*Drosophila* showed a preference for WE over NA strain after fermentation.

A series of preference assays was performed to test the feasibility of the fly behavioral preference assay in the T-maze. The flies showed a significant preference for cornmeal food over water, and no preference for fly food, water, or grape juice when these were placed in either arm of the T-maze (Supplementary Fig. 2b). The residual sugar and alcohol strength of six fermentates were measured by a refractometer and a vinometer, respectively, and revealed no significant differences, suggesting the impact of residual sugar or ethanol could be neutralized (Supplementary Fig. 3, a and b).

**Fig. 1.**
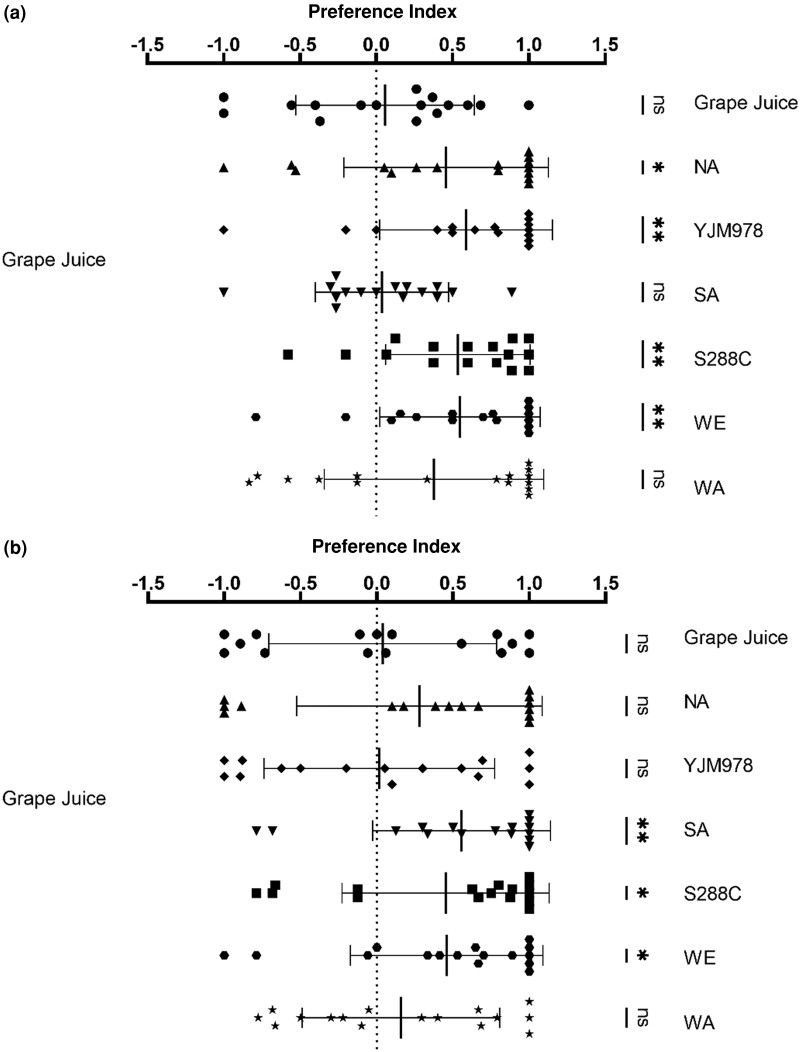
Preference assays of *Drosophila* fertilized females a) and males b) challenged with fermented (right-hand side) vs nonfermented grape juice. Mean ± SD preferences are shown for 16 individual trials. The right-hand column shows the results of one-sample *t*-tests compared with theoretical values: * *P* < 0.05, ** *P* < 0.01.

Twenty male and fertilized female flies were challenged with nonfermented or yeast-fermented grape juice for preference assay (Fig. 1, a and b). The comparison between two grape juices is to test the balance of the apparatus. Male and female flies shared a similar preference for WE (mean ± SD of PI female = 0.55 ± 0.53, *P* = 0.002; one-sample *t*-test; PI male = 0.46 ± 0.63, *P* = 0.01) and S288C strains (PI female = 0.54 ± 0.47, *P* = 0.001; PI male = 0.45 ± 0.68, *P* = 0.018). Although a similar directional response for NA was observed in males and females, the female response was significant (PI = 0.49 ± 0.67, *P* = 0.031), while the male response was weaker and not significant (PI = 0.28 ± 0.80, *P* = 0.185). Females showed a strong preference for YJM978 (PI = 0.59 ± 0.57, *P* = 0.002), while the males were neutral toward it (PI = 0.02 ± 0.76, *P* = 0.931); the opposite was observed for the SA strain (PI female = 0.04 ± 0.44, *P* = 0.856; PI male = 0.56 ± 0.58, *P* = 0.002). These results indicated that flies generally find fermented products more attractive than nonfermented grape juice.

**Fig. 2.**
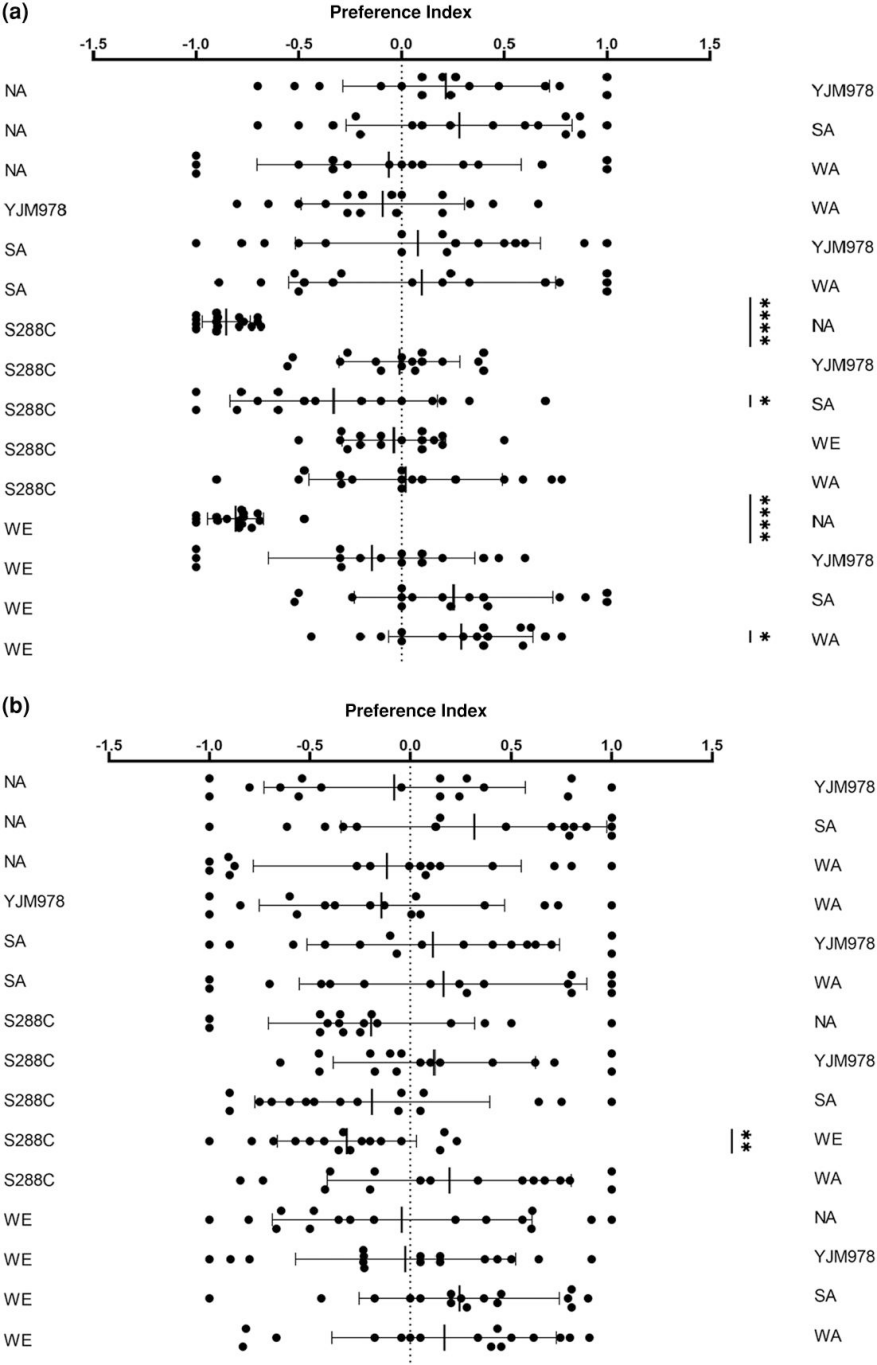
Pairwise comparison tests between fermentations. Mean ± SD preferences are shown for 16 individual trials. The right-hand column shows the results of one-sample *t*-tests: * *P* < 0.05, ** *P* < 0.01, **** *P* < 0.0001. a) Female and b) male.

We performed pairwise comparisons between the strains. Females showed a very strong preference for S288C (PI = −0.85 ± 0.12, *P* < 0.0001) and WE (PI = −0.81 ± 0.14, *P* < 0.0001) over NA. Females did not prefer S288C or WE (Fig. 2a), while males showed the modest preference for S228C over WE (PI = −0.32 ± 0.35, *P* = 0.002, Fig. 2b), revealing clear sex-specific differences in these behavioral responses.

### Strains from different origins show distinct volatile profiles

**Fig. 3.**
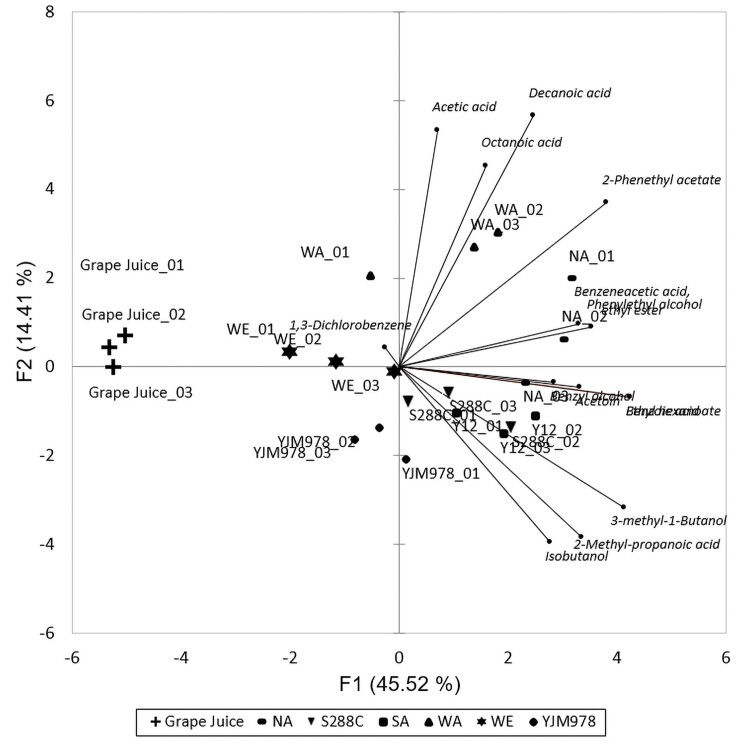
Principle component analysis of the volatile compounds in six yeast strains. The six strains and grape juice are separated by different symbols. Three dots with the same symbol represent three individual repeats. Small dots and italic words represent the quantified compounds. The lines and directions show correlations between variables and principal factors.

**Fig. 4.**
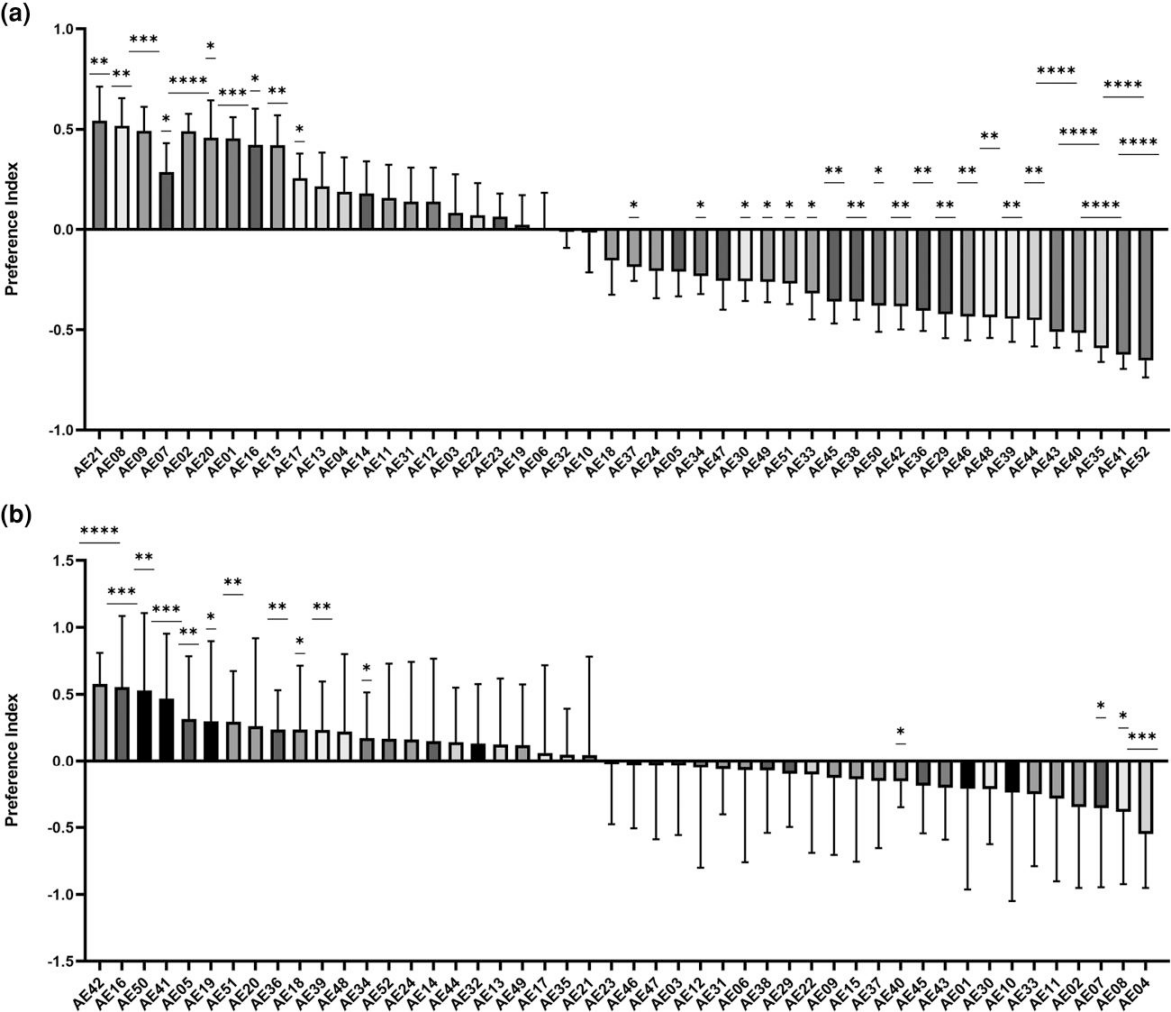
Female fly preference indices for F1 yeast progeny vs each parental strain. The positive values represent flies that preferred the fermented smell from the segregant, whereas the negative value was for the parental strain. Each bar represents the mean of preference indices. Segregants are sorted from the highest preference index to the lowest. Mean ± SD preferences are shown for 16 individual trials. The right-hand column shows the results of one-sample *t*-test: * *P* < 0.05, ** *P* < 0.01, *** *P* < 0.001, **** *P* < 0.0001. a) Segregants vs WE; b) segregants vs NA.

The construction of the GC–MS library was based on preliminary screening of the six yeast strains, diluted grape juice, and 24 F1 progeny of WE × NA used in the previous section. The aim of using volatile profile from segregants is to build a more comprehensive library. The compound list was refined by cross-comparison with the NIST11 database. The identities of 14 compounds were confirmed using commercial standards and quantified using a calibration curve (Supplementary Table 1 and Fig. 4). The choice of quantified compounds is based on the variation range of signal intensities from GC–MS. The quantified concentrations produced by all wild-type strains were analyzed using PCA. From the figure, the profile of WE is distinct from that of NA. The major compounds are ethyl phenylacetate (benzeneacetic acid, ethyl ester) and phenethyl alcohol. The compounds discriminating WA strains with others are benzoic acid, decanoic acid, and acetic acid. The character of Y12 and S288C is quite close, which is rich of isobutanol (2-methyl-1-propanol), isoamyl alcohol (3-methyl-1-butanol), and isobutyric acid (2-methyl-propanoic acid). Figure 3 shows seven groups distinguished by their compounds in PCA, indicating that the GC–MS profiles of the volatiles of the six yeast strains and grape juice differed. Based on the previous preference assay, flies can distinguish the fermented smell of WE and NA. Also, the PCA plot illustrated that WE and NA have distinct characteristics. Hence, these two strains were selected as the parental strains for further experiments. We selected the GC–MS data of these two strains for comparison. The concentrations of the 14 compounds quantified from WE and NA are listed in Table 1.

**Table 1.** Concentrations of the compounds from the fermentation of WE and NA strains measured using gas chromatography–mass spectrometry.

Name of compound	Concentration of WE (parts-per million, ppm)	Concentration of NA (ppm)	WE/NA (100%)
Benzoic acid	178.730 ± 15.711	228.628 ± 7.724	78.175
Ethyl phenylacetate	0.157 ± 0.019	0.299 ± 0.039	52.489
Acetoin	1402.539 ± 133.027	2283.866 ± 269.349	61.411
2-Phenethyl acetate	0.358 ± 0.065	1.029 ± 0.109	34.817
Isobutanol	70.821 ± 19.500	63.713 ± 5.549	111.156
Isoamyl alcohol	30.497 ± 10.120	61.929 ± 7.169	49.246
Phenethyl alcohol	28.207 ± 2.446	116.700 ± 12.273	24.171
Acetic acid	1516.343 ± 47.473	1506.944 ± 293.948	100.624
Ethyl hexanoate	0.001 ± 0.001	0.005 ± 0.004	28.581
1,3-Dichlorobenzene	0.141 ± 0.102	0.042 ± 0.002	339.982
Benzyl alcohol	0.114 ± 0.004	0.104 ± 0.010	109.038
Decanoic acid	4.465 ± 0.181	4.758 ± 0.444	93.851
Octanoic acid	12.439 ± 3.375	10.651 ± 4.223	116.790
Isobutyric acid	43.148 ± 2.184	35.568 ± 4.233	121.310

Concentrations are represented in mean ± SD (*n* = 3). The ratios are represented in mean_WE_/mean_NA_ × 100%.

WE, Wine European; NA, North America.

### F1 progeny of Wine European × North America exhibited a wide range of phenotypic variation

Female fruit flies can choose between WE and NA. This variation was further investigated using QTL mapping with the F1 progeny of WE and NA, previously generated by Cubillos *et al*. (2013), as the mapping population. We performed a preference assay for 48 segregants against each parental strain separately (Fig. 4, a and b). The reason for using the parental strains to perform comparisons is that the range of variation using nonfermented grape juice is relatively small. Flies preferred to choose fermented odors, from which preference indices cannot be used for QTL mapping. The preference indices elicited by segregants challenged with parental strains were categorized into nine groups (Table 2). Groups 1 and 9 reflect opposite phenotypes, in which the former (represented by segregant AE16) is repellant, whereas the latter (represented by AE40) is attractive compared with both parental strains. Groups 4 and 7, which include the majority of the segregants, included strains that do not elicit any significant preferences or elicit preferences for WE, but none for NA. The other groups showed various combinations of elicited preferences.

**Table 2.** Preference indices of segregants are categorized into groups depending on their mean and significance.

Group	Character of preference indices		ID of segregants
	WE	NA	
1	+	+	AE16
2	+	ns	AE01, AE09, AE15, AE17, AE20, AE21
3	+	−	AE02, AE07, AE08
4	ns	ns	AE03, AE06, AE10, AE11, AE12, AE13, AE14, AE18, AE19, AE22, AE23, AE24, AE31, AE32
5	ns	−	AE04
6	ns	+	AE05
7	−	ns	AE29, AE30, AE33, AE34, AE35, AE37, AE38, AE43, AE44, AE45, AE46, AE47, AE48, AE49, AE52
8	−	+	AE36, AE39, AE41, AE42, AE50, AE51
9	−	−	AE40

The volatile compounds of 92 F1 segregant fermentates were measured and analyzed. The concentrations of the volatile compounds among the segregants were compared individually (Supplementary Fig. 5). Fourteen individual graphs show the distribution of volatile segregant concentration. The acetic acid concentration of the two parental strains was relatively lower than that of most segregants. This trend was also observed for ethyl phenylacetate and isobutyric acid. In contrast, most segregants generated less isoamyl alcohol than WE and NA. Almost half of the segregants had the same concentration of 1,3-dichlorobenzene; however, half of the segregants did not produce this compound. Moreover, a few transgressive segregants were observed at concentrations >40 ppb. The concentration of each volatile compound can be considered a quantitative trait.

### Key candidate genes were revealed by QTL mapping of different traits

The ShmooTL QTL package (https://github.com/gact/shmootl) provides a platform for linking quantitative traits to genetic variation data. Here, we used the preference indices of the F1 progeny and quantified concentrations as quantitative traits (Naseeb *et al*. 2021; Gyurchev *et al*. 2022). The preference indices of the 48 segregants between WE and NA were considered as two separate traits (Supplementary Fig. 6). The concentrations of the 14 compounds determined using GC–MS were considered as 14 individual traits (Supplementary Fig. 7). The mapping results are listed in Table 3. In the preference assay with the WE trait (Average_PI_WE), the interval was located on chromosome V, where the peak LOD was located at 53 centimorgan (cM). The results of the preference assay with the NA trait (Average_PI_NA) showed the interval on chromosome III, where the peak LOD was located at 108 cM. The mapping results for the 14 compounds are shown in Supplementary Fig. 7. Only eight compounds showed some genotype markers. After tracing back to the four compounds mapping the results of which were lacking, the variation in the ranges of concentrations was found not to be as significant as those of the other eight compounds, suggesting that the phenotypic variation among the segregants was mild. The selected candidate genes for each trait were compared with the GO terms and synthesis pathways of target compounds in the Kyoto Encyclopedia of Genes and Genomes database. The candidate genes selected that were in agreement with the results of GC–MS and the preference assay are listed in Table 4. One of the mapped intervals emerges from the preference assay against NA, and the concentration of phenylethyl alcohol implicates two genes, namely *FUB1* and *PTC6*.

**Table 3.** R/QTL-mapped candidate intervals.

Trait	QTL name	Chromosome	Peak LOD	Start (cM)	Peak (cM)	End (cM)
2-Phenethyl acetate	c07:0199000 (*SUT1*)	07	2.88	43.76	72.51	101.25
Acetic acid	c01:0191000 (*YAT1*)	01	2.81	88.43	119.08	119.08
	c02:0116000 (*YBL055C*)	02	2.47	0.00	38.99	91.47
Acetoin	c04:0496000 (*VPS54*)	04	2.45	168.49	248.66	260.27
	c04:1146000 (*YDR336W*)	04	3.66	407.77	432.92	457.12
	c05:0236000 (*SAH1*)	05	2.70	61.59	96.22	137.54
	c05:0464000 (*SCC4*)	05	2.50	154.77	179.96	201.80
Benzyl alcohol	c03:0200000 (*MAT*)	03	2.21	0.00	66.58	85.86
Ethyl phenylacetate	c07:0268000 (*RSM23*)	07	2.13	43.76	101.25	130.00
	c10:0726000 (*YJR154W*)	10	2.73	227.16	266.16	266.16
	c12:0197000 (*AAT2*)	12	2.77	69.30	101.89	143.20
	c13:0659000 (*VTI1*)	13	2.36	186.84	254.49	308.91
Ethyl hexanoate	c04:0279000 (*POL3*)	04	2.13	116.26	168.49	244.40
	c07:0848000 (*ERG1*)	07	2.09	211.59	257.90	292.53
	c09:0380000 (*GAT4*)	09	2.72	182.51	192.87	192.87
Octanoic acid	c04:0189000 (*NOP14*)	04	2.41	116.26	145.01	217.52
	c09:0038000 (*SUC2*)	09	2.10	0.00	0.00	39.00
	c10:0106000 (*ERG20*)	10	2.93	0.00	23.48	69.91
	c11:0578000 (*SIS2*)	11	2.06	239.88	283.64	290.31
	c13:0659000 (*VTI1*)	13	2.19	231.00	254.49	308.91
	c14:0539000 (*ALG11*)	14	2.22	202.27	234.87	255.12
Phenethyl alcohol	c03:0283000 (*CDC39*)	03	3.31	68.67	143.57	143.57
	c07:0199000 (*SUT1*)	07	2.30	43.76	72.51	130.00
	c09:0192000 (*YIL091C*)	09	2.77	57.71	84.63	149.92
			2.88	43.76	72.51	101.25
Average_PI_NA	c03:0251000 (*PAT1*)	03	1.57	9.00	108.00	130.97
Average_PI_WE	c06:0094000 (*FRS2*)	06	1.39	0.00	53.00	136.44

**Table 4.** Information on candidate genes.

Candidate gene	Trait	LOD score	Brief introduction of genes
*GAT1 (YFL021W)*	Average_PI_WE	1.39	Sequence-specific DNA-binding RNA polymerase II transcription factor involved in positive regulation of transcription by nitrogen catabolites
*BST1 (YFL025C)*	Average_PI_WE	1.39	Phosphatidylinositol deacylase involved in vesicle organization, ER-to-Golgi vesicle-mediated transport, GPI anchor metabolism, protein retention in the ER lumen, protein–lipid complex remodeling, and ER-associated ubiquitin-dependent protein catabolism
*YFL040W*	Average_PI_WE	1.39	Putative transmembrane transporter that localizes to vacuole and prospore membrane
*FUB1(YCR076C)*	Average_PI_NA; phenethyl alcohol	1.57; 3.31	Proteasome-binding protein whose biological role is unknown
*PTC6(YCR079W)*	Average_PI_NA; phenethyl alcohol	1.57; 3.31	Protein serine/threonine phosphatase involved in mitophagy and macroautophagy; enables [pyruvate dehydrogenase (lipoamide)] phosphatase activity
*ARI1(YGL157W)*	2-Phenethyl acetate	2.88	NADPH-dependent aldehyde reductase
*ADY2(YCR010C)*	benzyl alcohol	2.21	Ammonium and acetate transmembrane transporter involved in nitrogen utilization
*SAT4(YCR008W)*	Benzyl alcohol	2.21	Protein kinase involved in protein localization, protein lipoylation, cation homeostasis, G1/S transition of mitotic cell cycle, and regulation of iron–sulfur cluster assembly

### Reciprocal hemizygosity assay validated genes that impact the production of volatile compounds

We compared all candidate genes between the parental strains to identify changes in protein sequences and the parental alleles in the segregants (Supplementary Fig. 8 andTable 2). There were two candidate intervals containing four genes corresponding to several traits on chromosome III. Crossovers were not observed between the genes in these mapped intervals in the segregants, and therefore, the categorization represents the whole candidate interval. *ADY2* and *SAT4* are two candidate genes in one candidate interval that correlate with the concentration of benzyl alcohol. However, *SAT4* encodes three amino acid changes (T243S, C281G, and R600H) between the two parental alleles, whereas *ADY2* encoded none, which was used as a negative control for further validation (Supplementary Table 2). In another interval on chromosome III correlating with the concentration of phenethyl alcohol and preference indices against NA, *PTC6* encoded two and *FUB1* four amino acid differences between the two alleles.

**Fig. 5.**
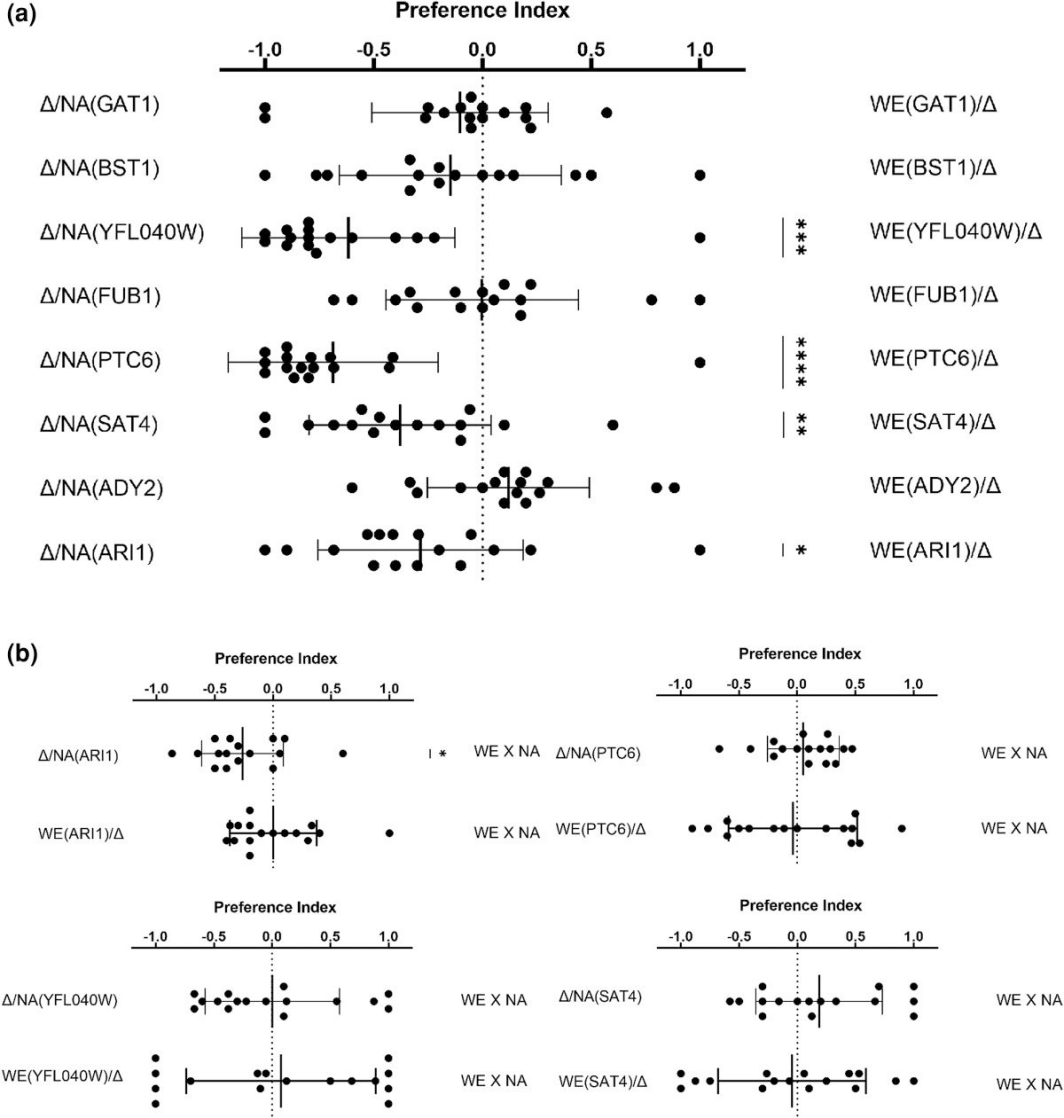
Preference from reciprocal hemizygosity assay. Comparisons are labeled on each side. Vertical line represents mean and horizontal error bars, SD (*n* = 16). * *P* < 0.05, ** *P* < 0.01, *** *P* < 0.001, **** *P* < 0.0001 in one-sample *t*-tests. a) Pairwise comparisons between each pair of hemizygotes. b) Comparisons between the four sets of hemizygotes that showed significant difference in (a) and the heterozygote strain.

The RHA directly compares the impact of different alleles on a phenotype (Liti and Louis 2012). Hybrids were constructed by independently knocking out one allele of the target gene at a time in WE and NA and then creating isogenic diploids with one or another allele. A head-to-head preference assay was performed to compare the two hemizygotes for each candidate gene (Fig. 5a). Four pairs of comparisons showed significant differences: *SAT4*, *PTC6*, *YFL040W*, and *ARI1*. All these hemizygotes carry the alleles from NA and are more attractive for flies than hemizygotes carrying WE alleles. As shown in Fig. 2a, the comparisons of the parental strains showed that flies preferred WE over NA. Consequently, the causative genes represent antagonistic QTLs (alleles, the effect of which differed from that of their parental origin), suggesting that the NA allele interacts with other genes of WE to produce the preferred volatiles. The success of RHA validation suggested that the PI can be used for QTL mapping. We also compared the preference between hemizygotes for the four causative genes and the heterozygote (Fig. 5b). Only hemizygotes carrying the NA allele of *ARI1* were preferred by flies over heterozygotes.

**Fig. 6.**
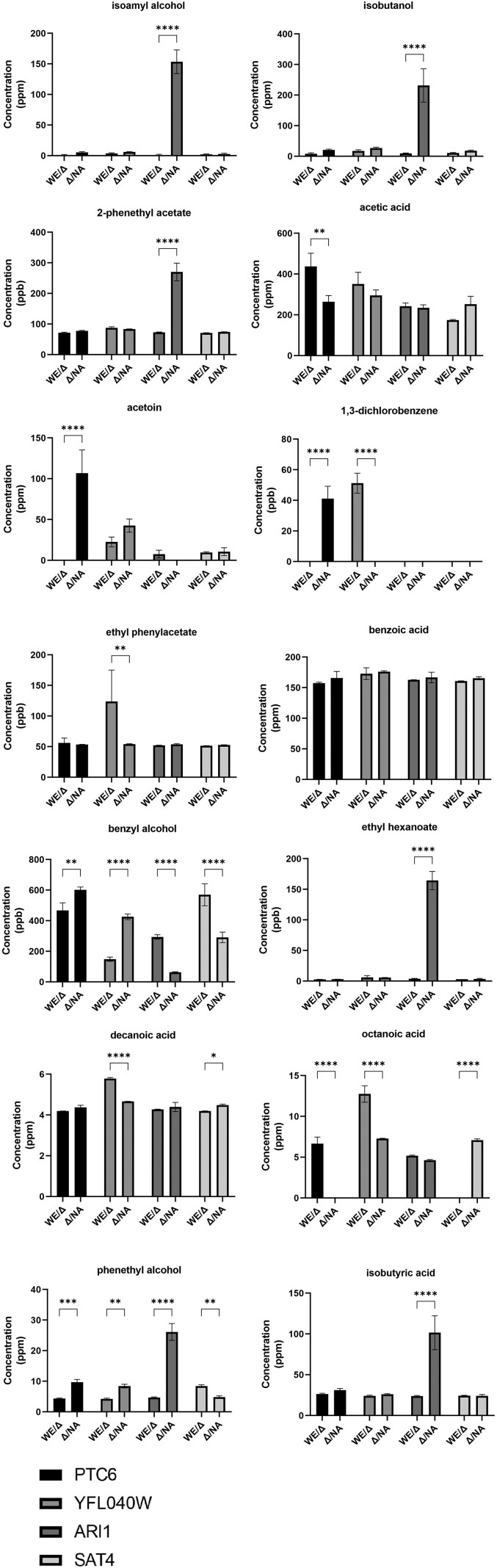
GC-MS of 14 compounds from four reciprocal hemizygous and heterozygous yeast strains. Each color represents the product of one gene, and both alleles are represented. Mean ± SD (*n* = 3). * *P* < 0.05, ** *P* < 0.01, *** *P* < 0.001, **** *P* < 0.0001 from *t*-tests.

To further decode the preferences elicited by the hemizygotes, the RHA strains of the four causative genes were analyzed using GC–MS, and the concentrations of the 14 compounds were quantified (Fig. 6). The concentration of benzoic acid did not vary among the hemizygotes, whereas the two hemizygotes for *PTC6* produced different concentrations of acetic acid, acetoin, phenethyl alcohol, 1,3-dichlorobenzene, benzyl alcohol, and octanoic acid. Except for acetic acid and octanoic acid, the hemizygotes carrying the NA allele produced a higher concentration of the other compounds than the WE-carrying hemizygotes. This suggests that different alleles of *PTC6* differentially affect a series of compounds and fly preferences. The allelic effects of *YFL040W* were observed on the levels of ethyl phenylacetate, phenethyl alcohol, 1,3-dichlorobenzene, benzyl alcohol, decanoic acid, and octanoic acid. Although the NA allele was associated with high alcohol levels, the hemizygote carrying *ARI1* from NA generated considerably high concentrations of isoamyl alcohol, isobutanol, 2-phenethyl acetate, ethyl hexanoate, phenethyl alcohol, and isobutyric acid, but not benzyl alcohol. The allelic differences correlated with phenethyl alcohol, benzyl alcohol, decanoic acid, and octanoic acid.

### Synthetic liquid preference assay validated the results from gas chromatography–mass spectrometry

**Fig. 7.**
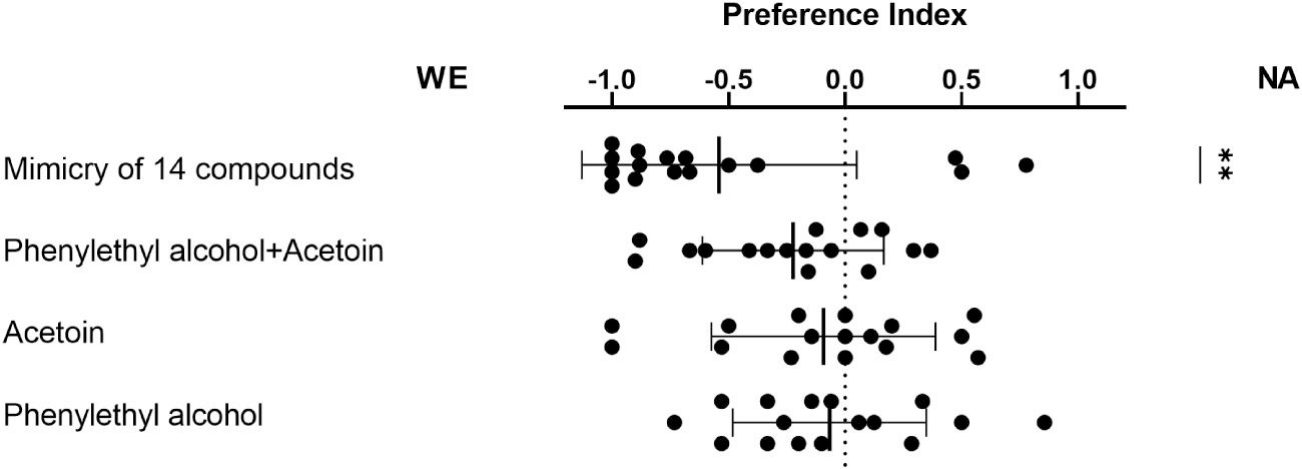
Results of the preference assay comparing artificial mixtures mimicking the concentrations measured using GC–MS. Left WE and right NA. Each comparison compares the differences between single or multiple compounds. Means and SDs are as in previous figures, *n* = 16. ** *P* < 0.01 using one-sample *t*-tests.

Further verification and investigation of the effects of the compounds on behavior were performed using preference assays in which artificial volatile mixtures that mimic the GC–MS constitution of WE and NA were compared. Comparison of a mixture of the 14 quantified compounds revealed a significant preference for the WE mixture (Fig. 7), which is consistent with the result of the original preference assay shown in Fig. 2a. The PCA results of GC–MS revealed few principal compounds that appear to be important for distinguishing fermented odors. The artificial parental mixture of phenethyl alcohol and acetoin did not elicit any significant preference, although 10 of the 16 data were in the direction of WE, as expected. Presentation of the individual components of this mixture did not elicit significant preferences. Consequently, it appears that the interactions among several compounds in the mixtures elicited olfactory preferences in female flies.

## Discussion

The interaction between fruit flies and yeast, mediated by volatile compounds, represents a sophisticated mutualism that has evolved over thousands of years (Chakraborty *et al*. 2022). However, the studies relating to this interaction are still limited to *S. cerevisiae* and two *Drosophila* species (*Drosophila simulans* and *Drosophila melanogaster*). Palanca *et al*. (2013) report that not all *S. cerevisiae* strains showed attractiveness. Moreover, some yeasts from different genera (*Pichia kluyveri* and *Hanseniaspora uvarum*) and other *Saccharomyces* species (*Saccharomyces bayanus* and *Saccharomyces uvarum*) are found to be attractive to fruit flies, which could be more representative of the natural environment. We used *S. cerevisiae* in this study as a genetically controllable tool to locate potential causative gene variants.

Previous studies have suggested a strong ecological correlation between wild yeast strains and fruit flies sharing the same niche (Becher *et al*. 2012). Flies used in this study were captured in Italy (M1201), the yeast strain WE was obtained from a vineyard from France (Liti *et al*. 2009), and S288C was originally derived from strain EM93, which was isolated from a rotten fig in the USA (Mortimer and Johnston 1986). Sequence data show that the genome sequences of WE and S288C are closely related (Liti *et al*. 2009). It is reasonable to speculate the possibility that European flies might prefer European yeast. However, Quan and Eisen selected yeast strains and wild flies from the same micro-environment and assayed preference, oviposition, larval development, and longevity. The results found no specific correlation between flies and yeast from different micro-habitats (Quan and Eisen 2018). Future exploration could use flies and yeasts from different continents to perform comparisons and identify genes involved in odor preference.

The aroma from fermented substrates is a complex combination of volatile compounds. QTL mapping has been used to study the genetic basis of fermenting parameters, such as sugar concentration (Salinas *et al*. 2012), nitrogen consumption (Molinet *et al*. 2019), and sulfite production (Noble *et al*. 2015). One study used progeny of 4CAR1 and T73 strains exploring the QTL behind the volatile compounds (Eder *et al*. 2018). The results identified 23 volatile compounds from YPD-based fermentates and further validated 13 causative gene variants influencing various traits. The compounds identified show some overlap with our study, such as 2-phenethyl acetate and phenethyl alcohol. However, our study quantified the concentrations of acetic acid and acetoin. The difference of measured compounds might be due to the difference of substrate or the strains used. The 14 compounds chosen here and quantified are the most representative compounds from GC–MS. The signal intensities of these compounds vary significantly among the six wild yeast strains. Although there are numbers of other compounds existing in the aroma, further preference assays using standard compounds mimicking the composition of fermentation aroma validated the importance of these 14 compounds. In a next step, we would like to remove one of the 14 compounds to see the impact on the preference assay in order to explore which compound might be the dominant factor in the mixture. A previous study used nonfermented grape juice as a comparison in a preference assay (Palanca *et al*. 2013). This can discriminate if the attracting smell originates from fermentation. Becher *et al*. (2012) also mentioned that yeast is the main factor attracting fruit flies, rather than the fruit itself.

Epistasis is an unavoidable factor in complex trait analysis (Huang *et al*. 2012). There are three major possibilities for epistasis: dominant epistasis, co-adaptive epistasis, and dominance-by-dominance epistasis (Carlborg and Haley 2004). The QTL mapping used in this study is for individual QTLs, which is focused on detecting the genetic influence, rather than the interactions (Van Laere *et al*. 2003). The disadvantage of this approach is neglecting the impact of genetic background or interaction among loci (Ljungberg *et al*. 2004). Epistatic QTL mapping can provide mean effects of multi-locus genotypes on the phenotype. Consequently, the requirement for data quality and analytical algorithm is much higher than normal QTL mapping, which restricts the study of epistasis. Salinas *et al*. (2012), using a series of hemizygotes, validated epistatic effect between *FLX1* and *MDH2* alleles resulting in a high concentration of succinic acid. The cross-combination of our gene-knockout mutants, especially *PTC6* and *ARI1*, will be used for preference assays and volatile compound quantification to reveal whether there is epistasis.

One of our results above suggested that female flies have different preference indices compared with male flies. Female flies have the responsibility of reproduction, which is likely to be more sensitive to a nutritious environment (May *et al*. 2015). Sex differences have been studied in epigenetic regulation and gene expression (Jazin and Cahill 2010). In *Drosophila,* Sxl and Tra proteins are expressed sex-specifically in females (Jazin and Cahill 2010) and sex-specific forms of Fru (fruitless) and Dsx (doublesex) genes control courtship and aggression (Robinett *et al*. 2010; Rideout *et al*. 2010). These major sex determining genes will be mediating the sex differences from various perspectives. For example, an odorant receptor, *Or67d*, recognizes male *cis*-vaccenyl acetate (an aggregation and courtship inhibiting pheromone) and its projections show sex differences in their targets in the antennal lobe (Datta *et al*. 2008; Ruta *et al*. 2010). In the same way, the receptors for yeast odorants may be sex-specifically expressed in both sexes, but their neurogenetic wiring may differ.

Four causative genes, namely *PTC6*, *SAT4, YFL40W,* and *ARI1*, have been mapped in this study. The GC–MS data of hemizygotes revealed how allelic variation influences the production of volatile compounds. YFL040Wp is a putative transporter that localizes to the vacuole and cell membrane (Giaever *et al*. 2002) and is involved in transmembrane transport (Palma *et al*. 2007) and production of acetic acid and glycerol (Salinas *et al*. 2013). SAT4p kinase is involved in protein localization, protein lipoylation, cation homeostasis, G1/S transition of the mitotic cell cycle, and regulation of iron–sulfur cluster assembly (Mulet *et al*. 1999; Gey *et al*. 2014). One study reported that the overexpression of SAT4p reduced the levels of Fe–S-containing proteins (ACO1p, LYS4p) and the activities of mitochondrial aconitase, α-ketoglutarate dehydrogenase, and pyruvate dehydrogenase complex (PDC) (Gey *et al*. 2014). The mitochondrial role of SAT4p is also supported by the fact that it interacted with the mitochondrial C1-tetrahydrofolate synthase, MIS1p (Ashton and Hickson 2010 ), which is phosphorylated on S355 (Vlastaridis *et al*. 2017). The involvement of SAT4p in MIS1p phosphorylation, as well as the functional significance of their interaction, remains to be tested. Interestingly, negative genetic interactions between *SAT4* and *YCK2* (yeast casein kinase) have been described (Sharifpoor *et al*. 2012; Costanzo *et al*. 2016); however, these could be the result of SAT4p's involvement in regulating the stabilization of transporters in the plasma membrane (Mulet *et al*. 1999). In this study, *SAT4* affected the concentrations of benzyl alcohol, phenethyl alcohol, and octanoic acid, although the mechanism remains elusive. One possible interpretation is that SAT4p interacts with LAT1p [dihydrolipoamide acetyltransferase component (E2) of the PDC] to influence the activity of pyruvate dehydrogenase in the citric acid cycle, further affecting the production of secondary metabolites (Gey *et al*. 2014).

PTC6p is a mitochondrial type 2C protein phosphatase (PP2C) associated with EC 1.2.4.1 pyruvate dehydrogenase activity in *S. cerevisiae* (Ruan *et al*. 2007). Its main function is to dephosphorylate PDA1p and PDB1p, which are core enzymes involved in pyruvate metabolism (Gey *et al*. 2008). Loss of *PTC6* reduces PDC activity, concomitant with elevated PDC phosphorylation (Guo *et al*. 2017). In fact, deletion of *PTC6* can result in hyperphosphorylation PDA1p (González *et al*. 2013). Metabolomics analysis has suggested that the *PTC6Δ* strain has significantly higher free valine, alanine, and isoleucine concentrations than the wild type (Mülleder *et al*. 2016). In the Ehrlich pathway, l-valine is responsible for producing isobutanol and isobutyrate, and l-isoleucine can produce active amyl alcohol and methylvalerate (Ebert *et al*. 2017). The two different alleles in our study affected the levels of acids, ketones, and alcohols, suggesting that pyruvate metabolism is important for the production of secondary metabolites. However, the allelic effect of *PTC6* on the production of volatile compounds remains unclear. Hence, investigating the allelic impact on phosphorylation of EC 1.2.4.1 or other proteins as substrates would be useful.

*ARI1* encodes an NADPH-dependent aldehyde reductase that utilizes aromatic and aliphatic aldehyde substrates for the reduction of aldehydes to alcohols in the presence of NADPH (Kaniak *et al*. 2004; Liu and Moon 2009). The results of this study indicated that different alleles of *ARI1* can affect multiple volatile compounds, except for all acid compounds measured using GC–MS in the volatile profiling of hemizygotes. *ARI1* may influence the generation of alcohols via aldehyde reduction, further affecting the concentrations of some of the esters. ARI1p converts phenylacetaldehyde to phenethyl alcohol, which is synthesized from glucose via the Ehrlich pathway (Hazelwood *et al*. 2008). It is also involved in other biological processes, such as oxidoreductase activity and ethyl acetoacetate reduction activity (Moon and Liu 2015). We visualized the structural differences between the proteins encoded by each pair of alleles using AlphaFold (Jumper *et al*. 2021). The result of protein alignment revealed three amino acid positions, namely 145, 189, and 341, that varied between two alleles, have a potential impact on the protein structure (Supplementary Fig. 9). The AlphaFold prediction suggests that amino acid variation may influence the structure, indicating that structure–function relationships should be investigated.

In the wild environment, adult fruit flies aggregate around the food area, where normally courting, mating, and ovipositing also occur (Mueller *et al*. 1991). Initially, a group of flies will locate the most favorable food patch and then the remainder follow (Tinette *et al*. 2004) using their powerful olfactory system which can discriminate multiple discrete odorants (Vosshall 2000). It is possible that some flies may simply “follow the leader” to make the choice in our own experiment. This could be circumvented by performing individual fly experiments but would considerably reduce the statistical power of the experiment given the reduction in N. Another point we observed is the high variance of preference indices which would be reflected in the QTL mapping results where we may have overlooked other, important, but perhaps less statistically significant loci.

Our study is limited by the number and nature of the offspring from a cross between yeast strains whose fermentation volatile profile elicited opposing preferences in *Drosophila*. The 96 offspring studied were previously genotyped at a density of segregating markers that was greater than the number of crossovers expected per meiosis (Cubillos *et al*. 2011), yet our mapping resolution is limited to tens of centimorgans for the traits mapped. Higher-resolution mapping would require additional recombinant and/or additional generations of recombination, which was not feasible for this study given the number of assays required. As such, the QTL regions may contain more than one causal variant, and the candidate genes were chosen from among hundreds contained within a QTL peak. This study was not designed to explain all the genetic variation underlying attractiveness to flies but to demonstrate that attractiveness of fermentation volatiles is complex. The RHA does validate segregating variation as causal in altering volatile compound profiles. Proving these same genes impact attractiveness would require direct allele replacement in haploid strains (Liti and Louis 2012) and attractiveness assays using *Drosophila*. Despite its limitations, this study has identified variation at four genes that affect volatile composition which are also likely to impact *Drosophila* choice and have further demonstrated their complex effects including actions opposing expectations. Nevertheless, and in conclusion, this study locates four causative genes influencing the production of yeast volatiles that mediate attraction/repulsion of *Drosophila*. Our approach provides a method for analyzing similar yeast–fly interactions in other fly species of agricultural importance. It also can underpin the genetic dissection of the fly olfactory receptors or combination of receptors that determine the attractive and repellant behavioral responses.

## Supplementary Material

iyae048_Supplementary_Data

## Data Availability

The RHA strains are available upon request. The genotype data of F1 progeny can be found in Cubillos *et al*. (2011) (doi:10.1111/j.1365-294X.2011.05005.x). The ShmooTL R package is available at (https://github.com/gact/shmootl). Supplemental material available at GENETICS online.
