# Supplementary material for: Quantitative genetic analysis of attractiveness of yeast products to Drosophila
Source: Genetics. 2024 Apr 1;227(2):iyae048. doi: 10.1093/genetics/iyae048 (PMC11151935; doi:10.1093/genetics/iyae048)

## Supplementary data figures

Fig S1.


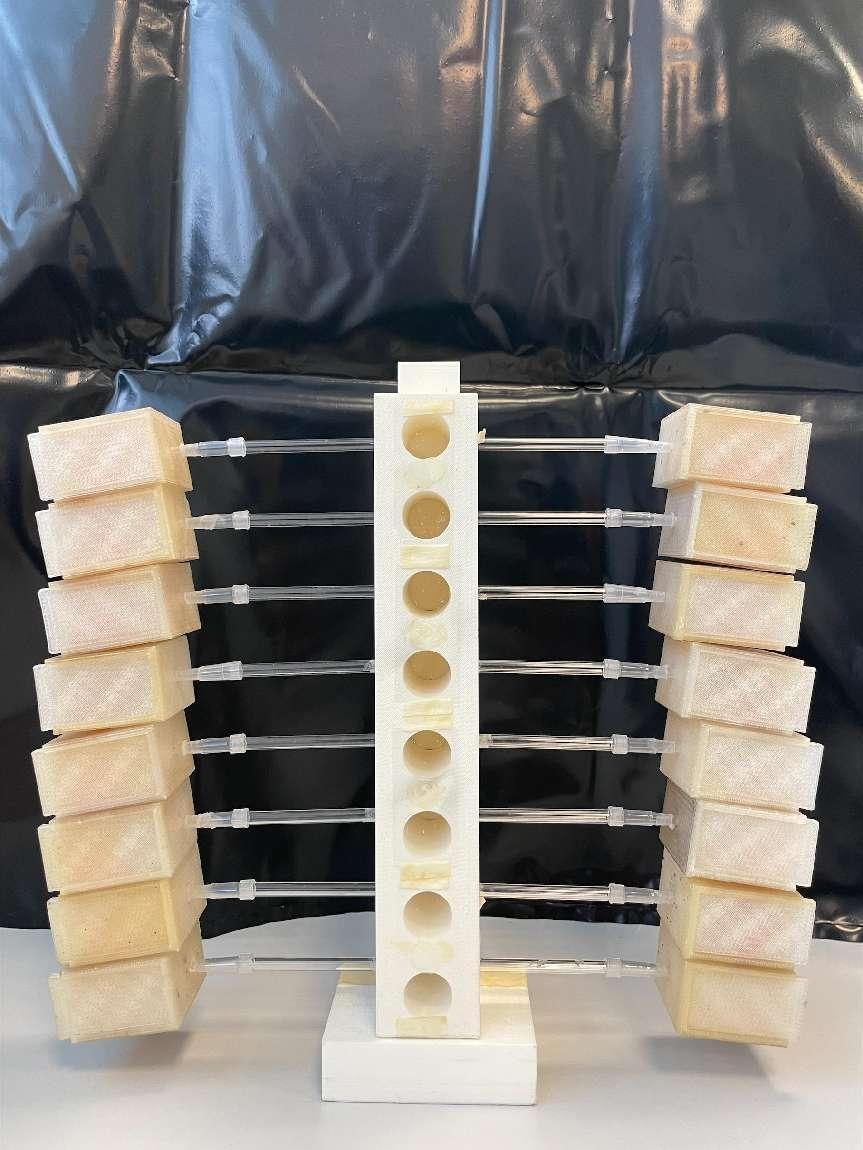


A


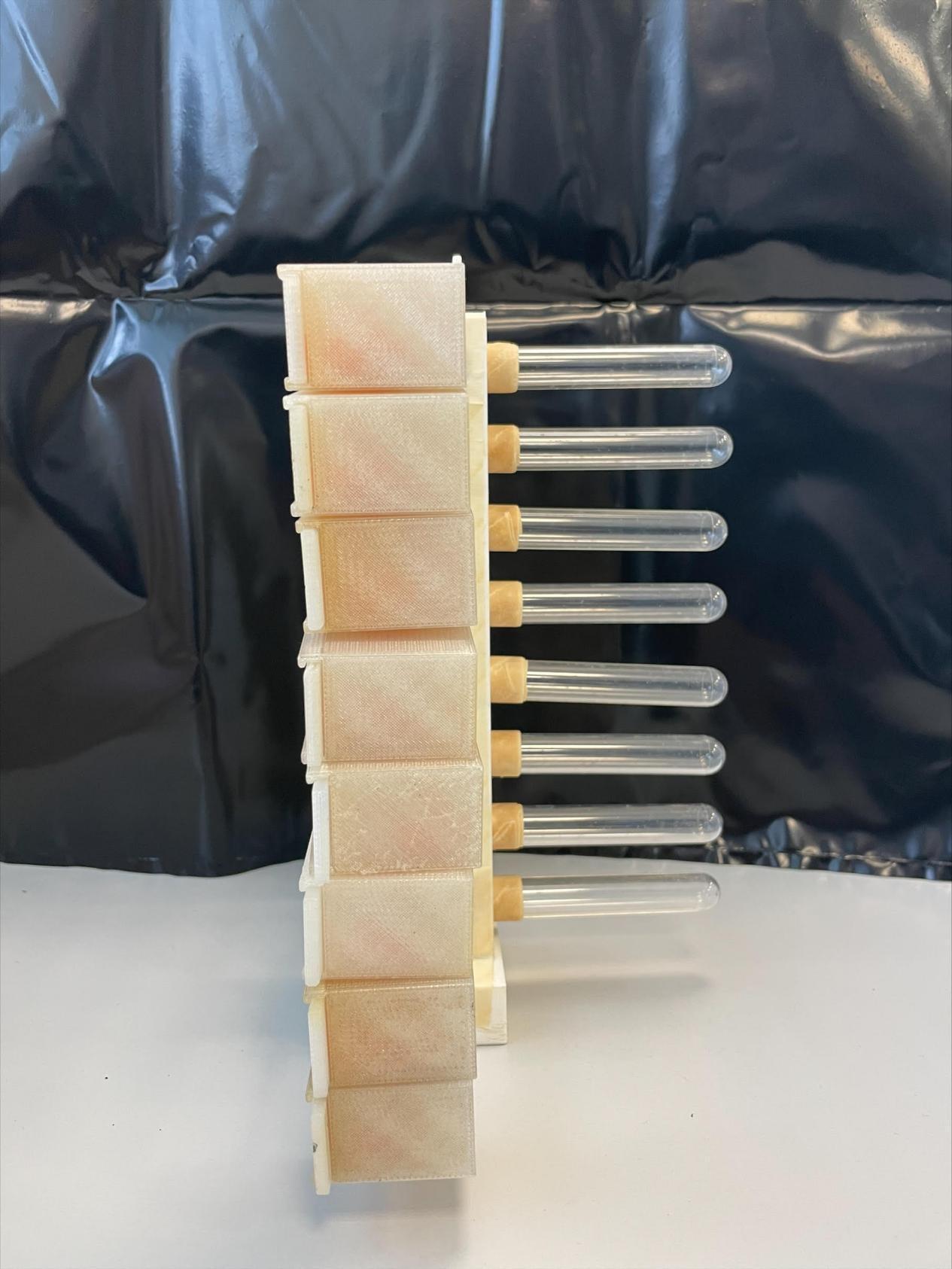


B


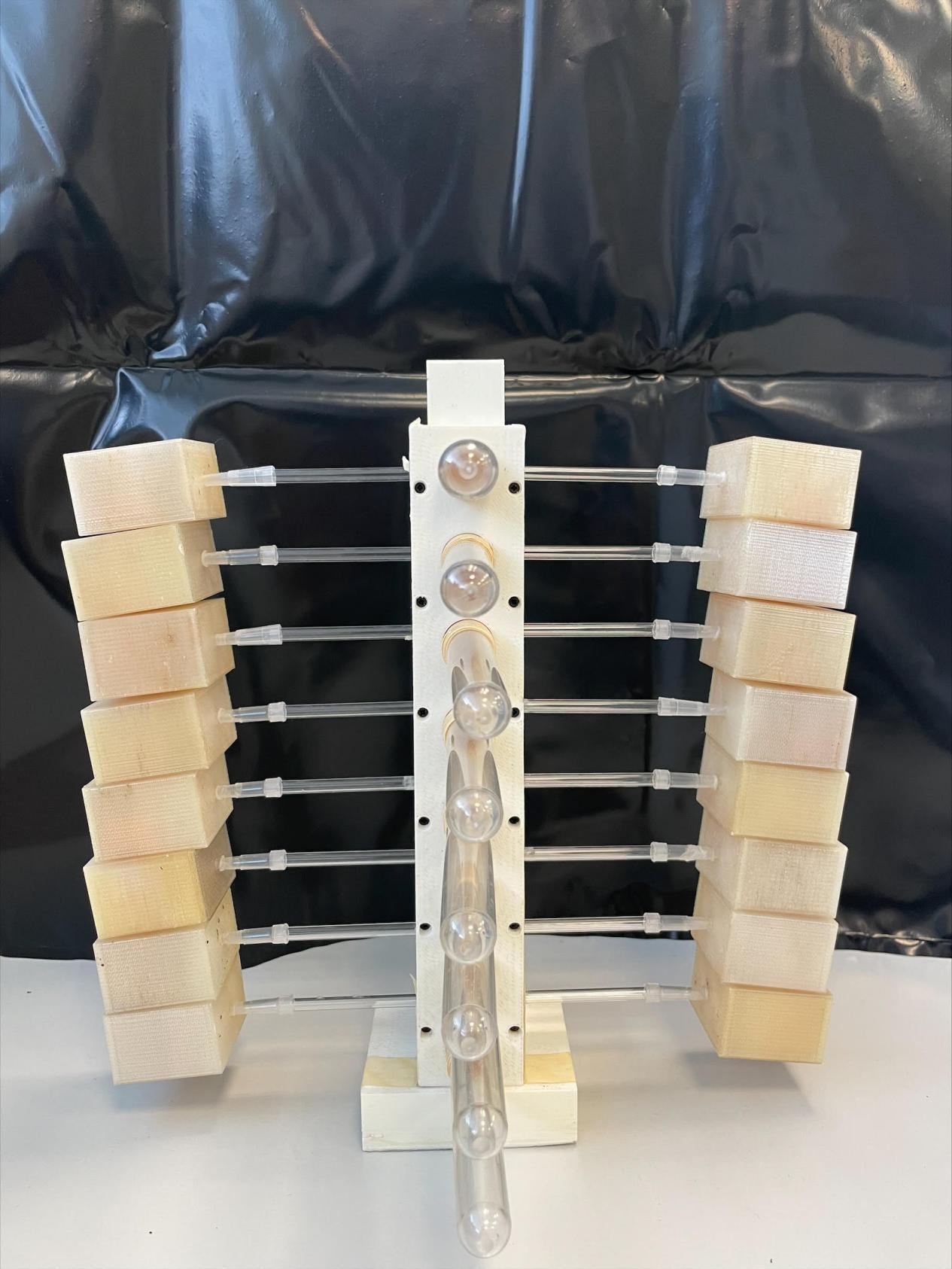


C


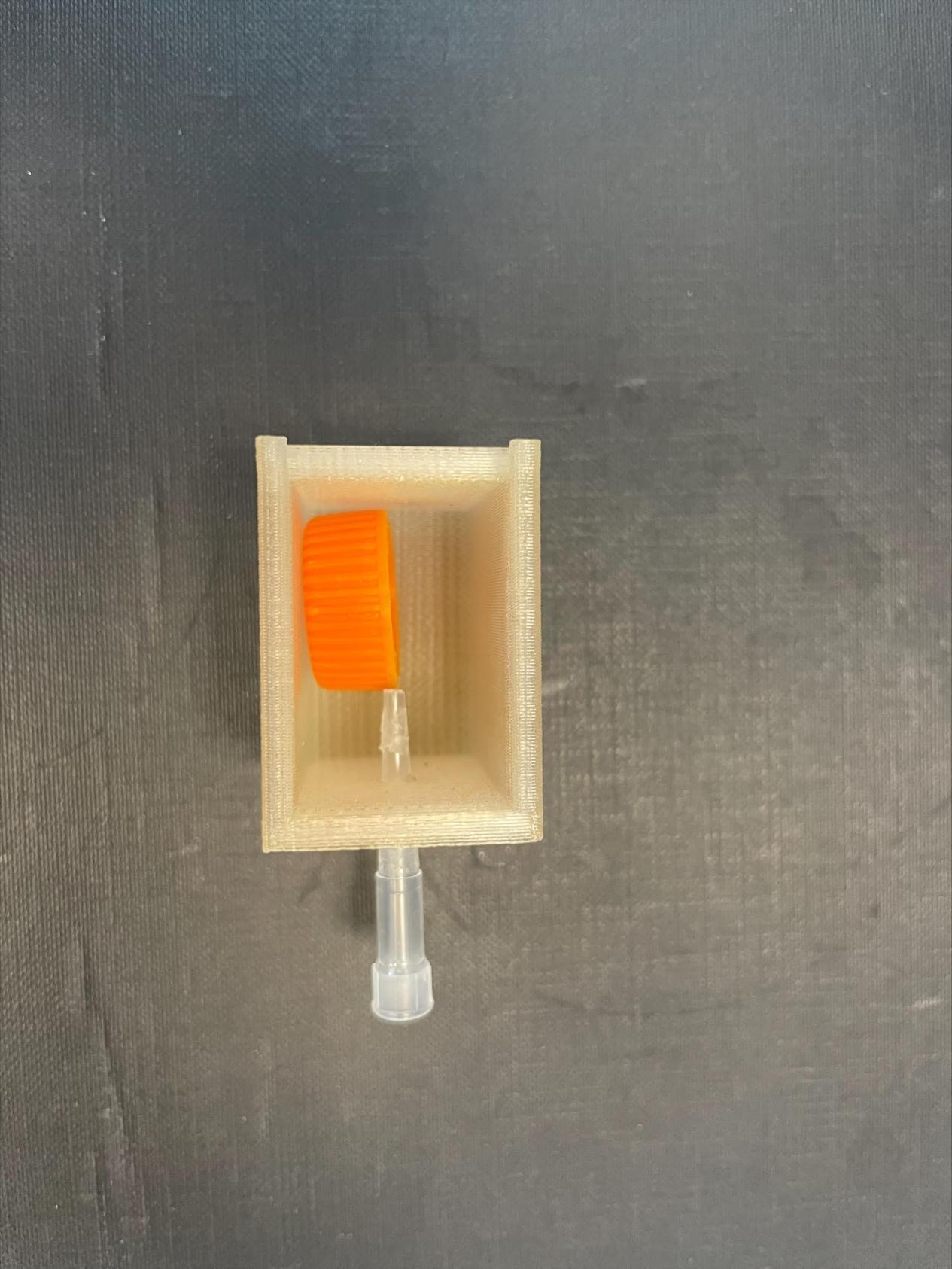


D

Fig S2.


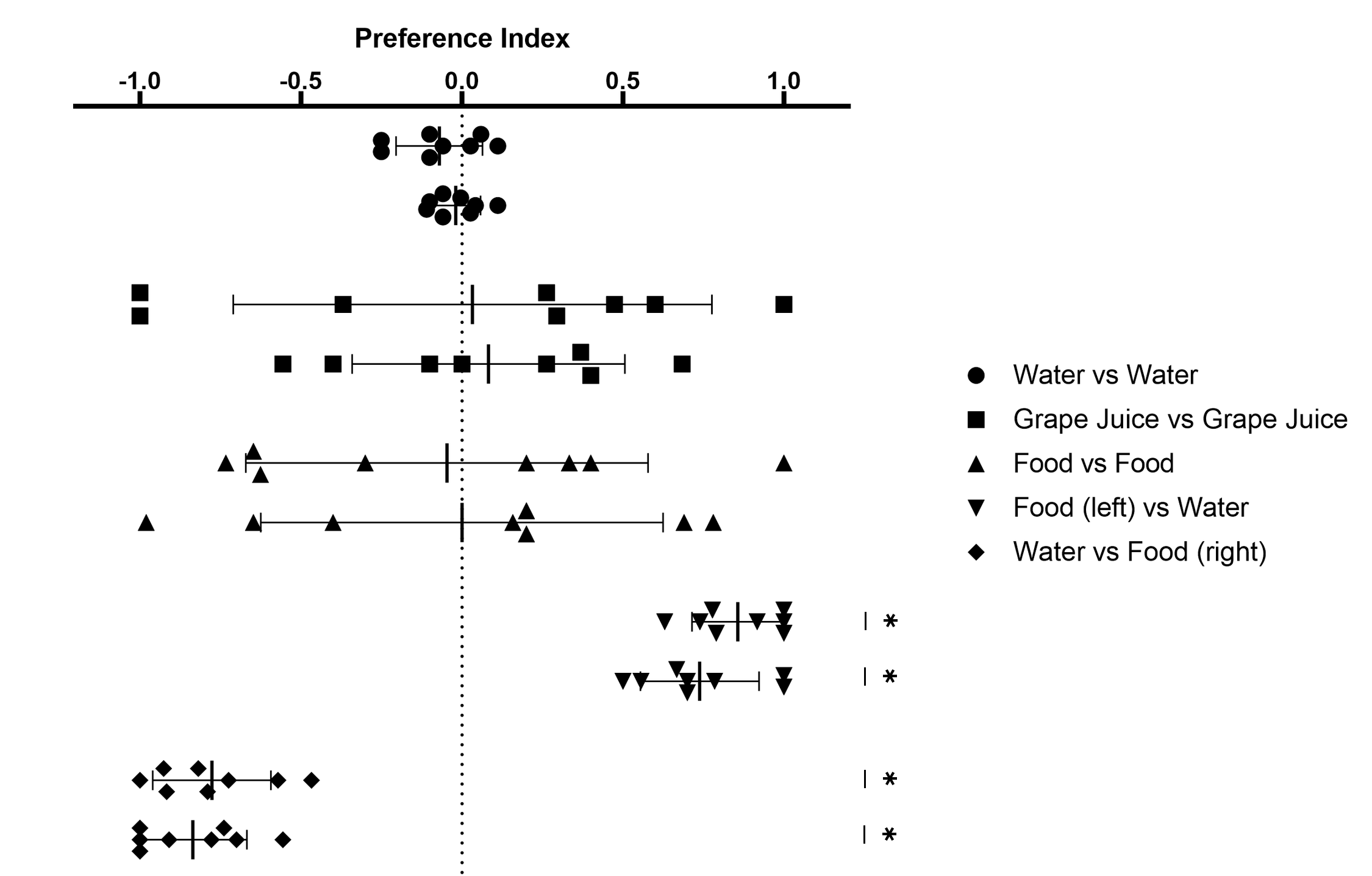


Fig S3 A.


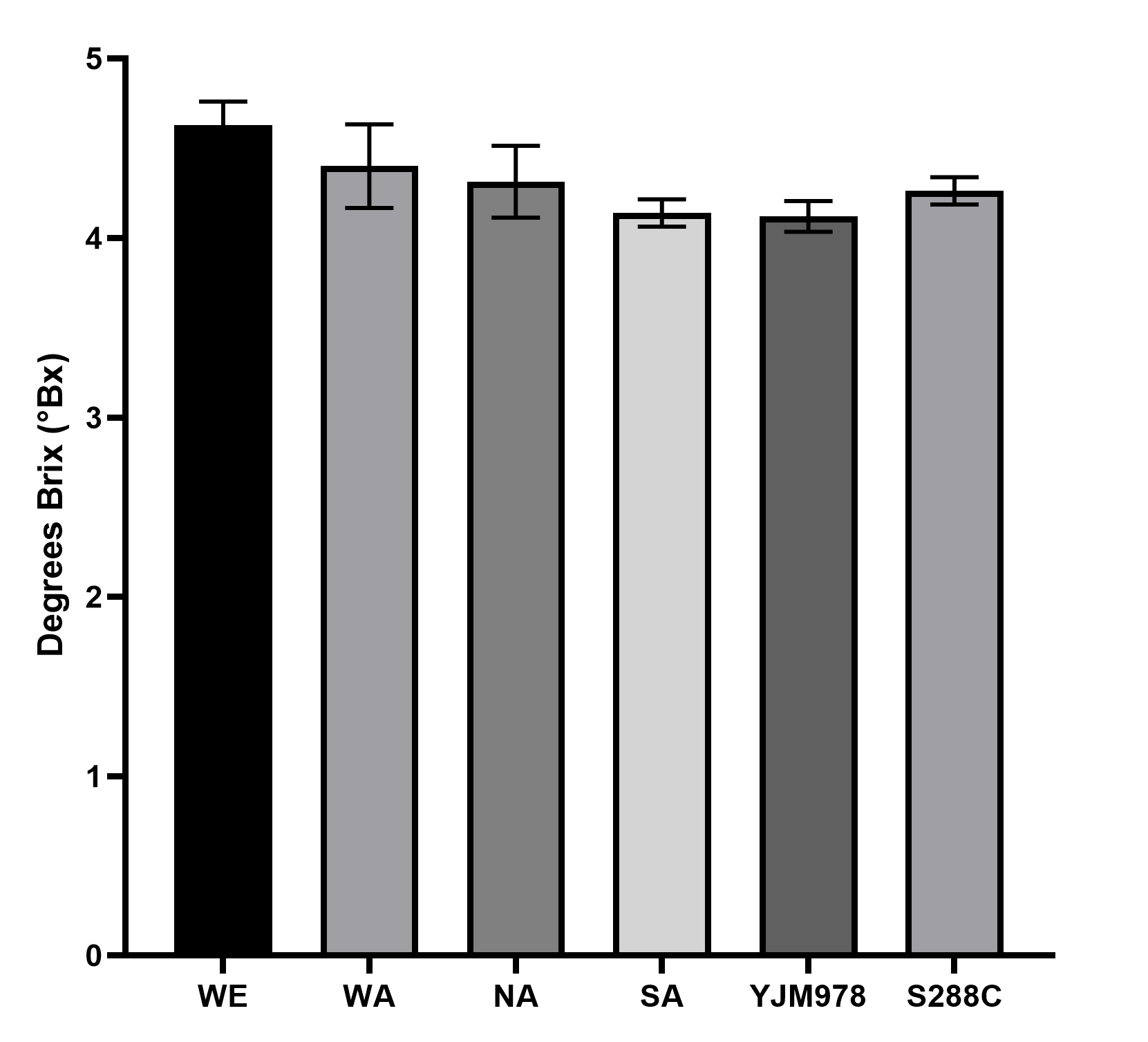


Fig S3 B.


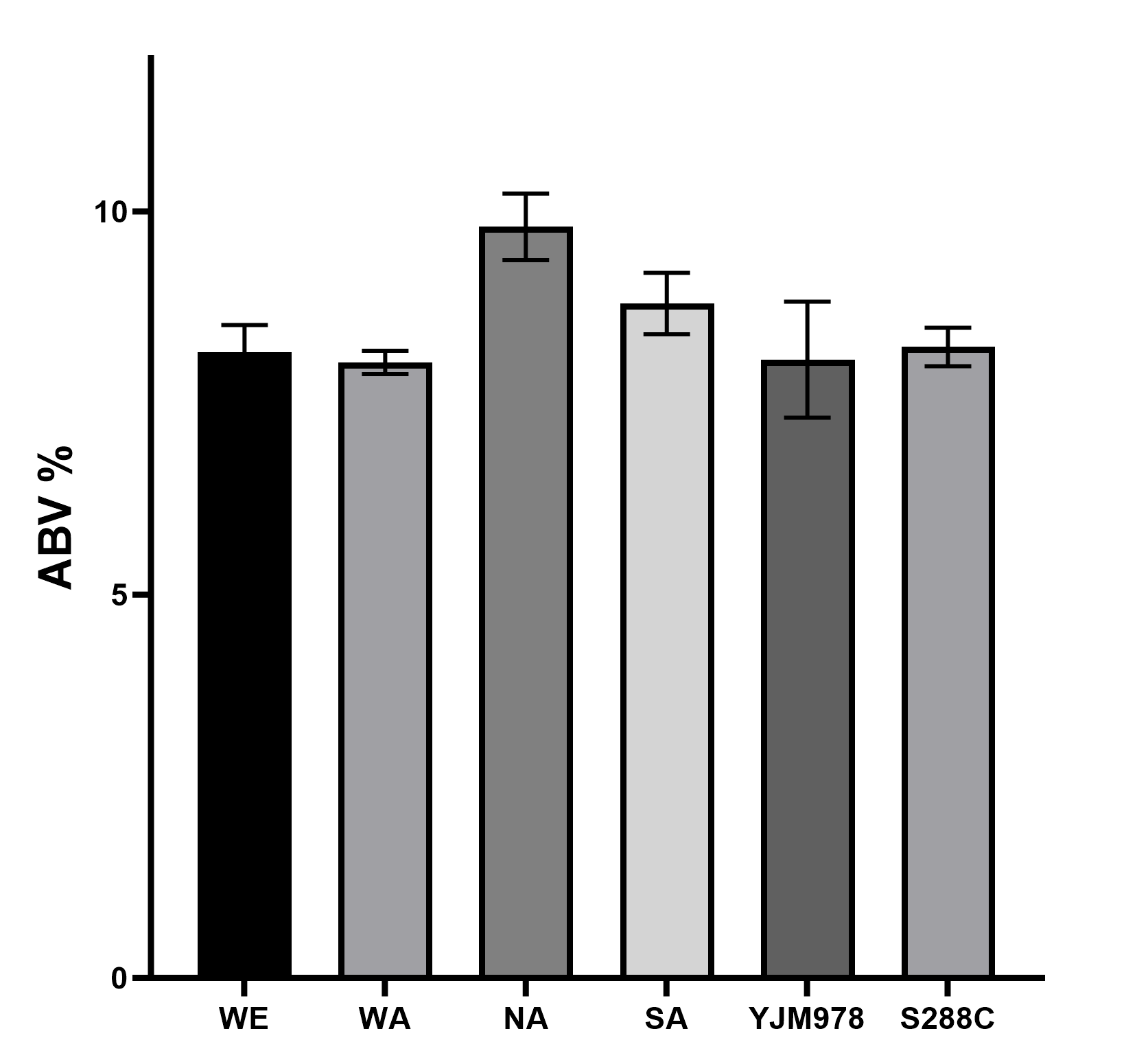


Fig S4.


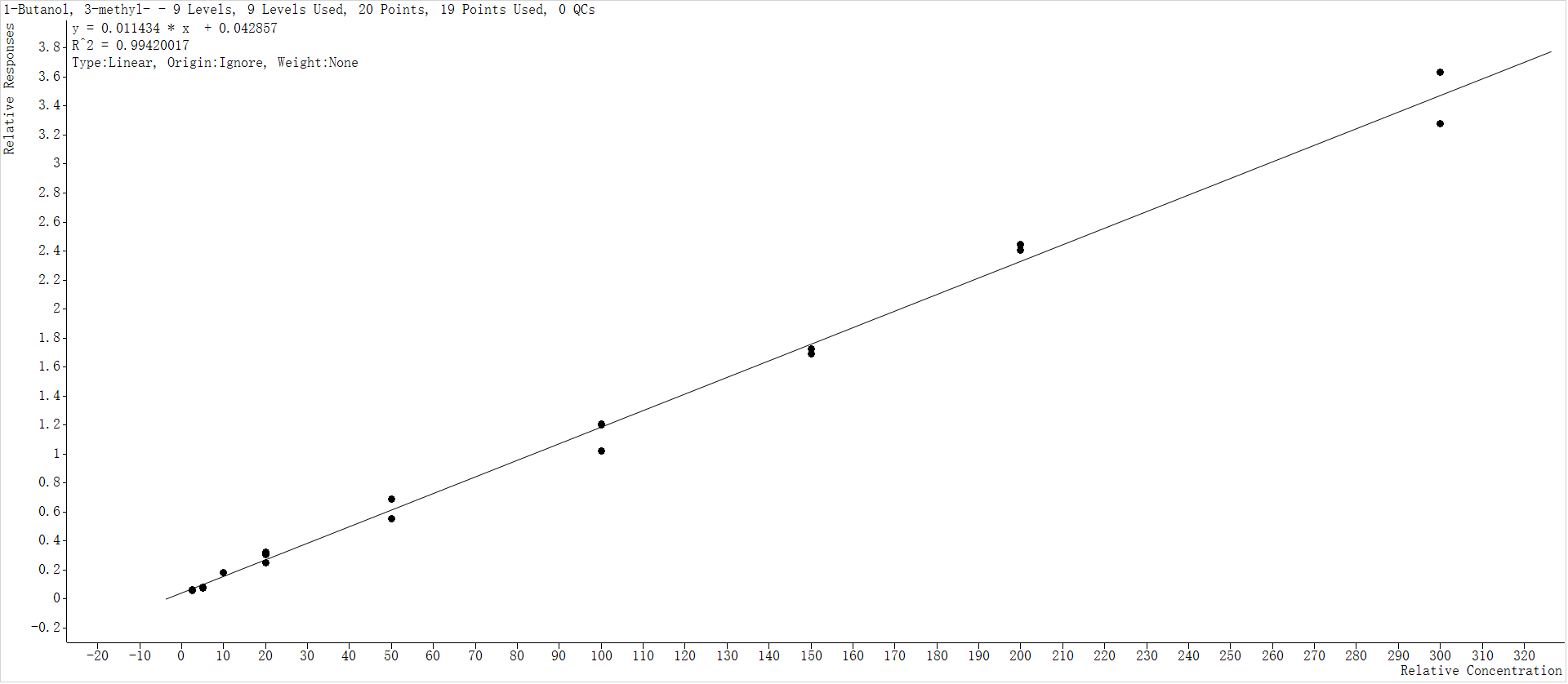


Isoamyl alcohol


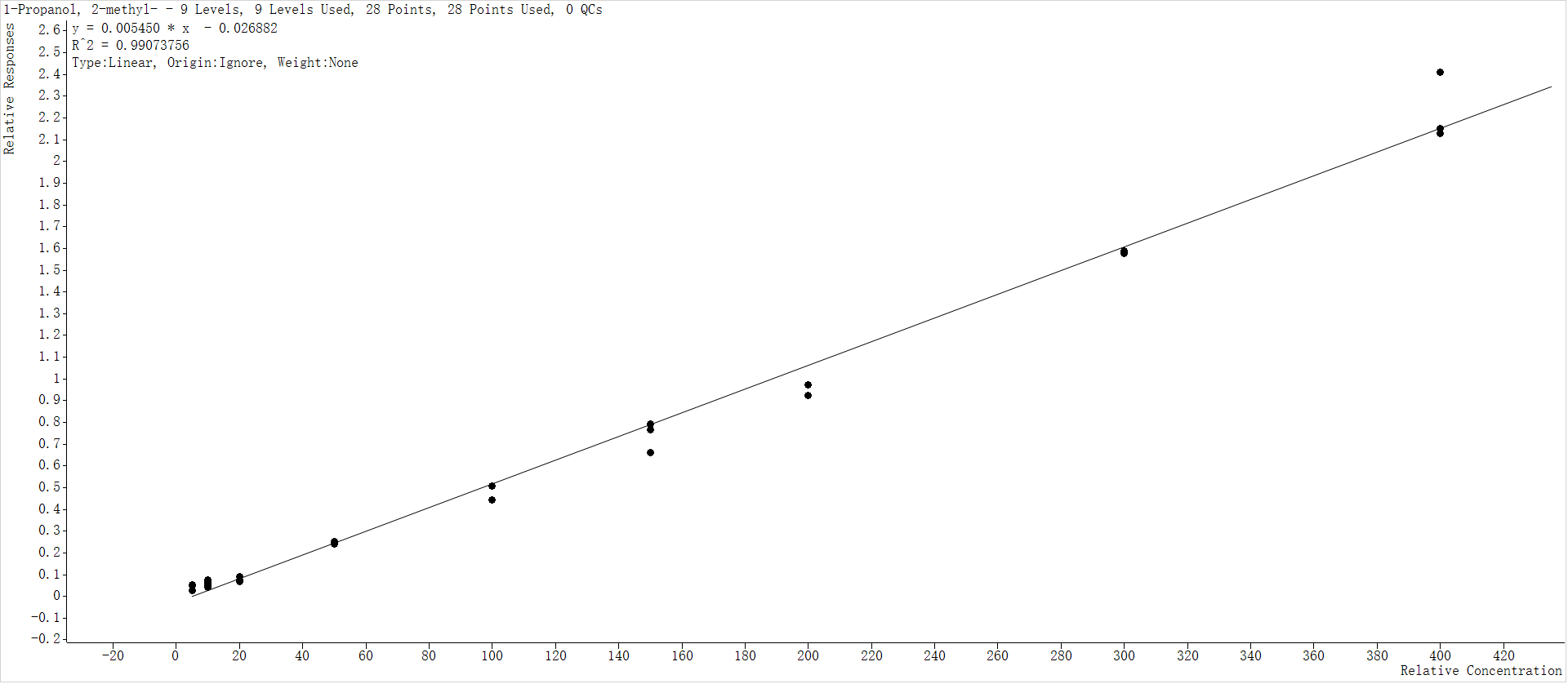
Isobutanol


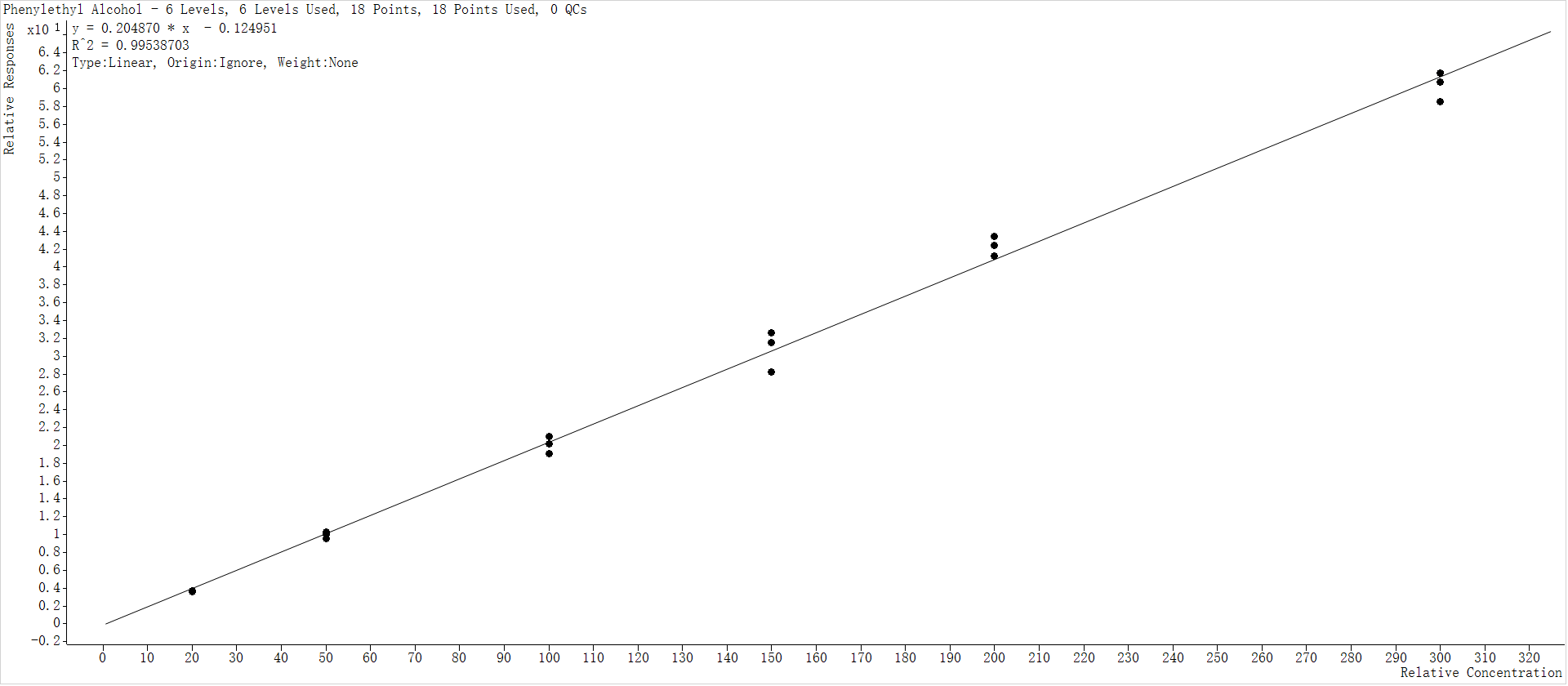
Phenethyl alcohol (for diploids)


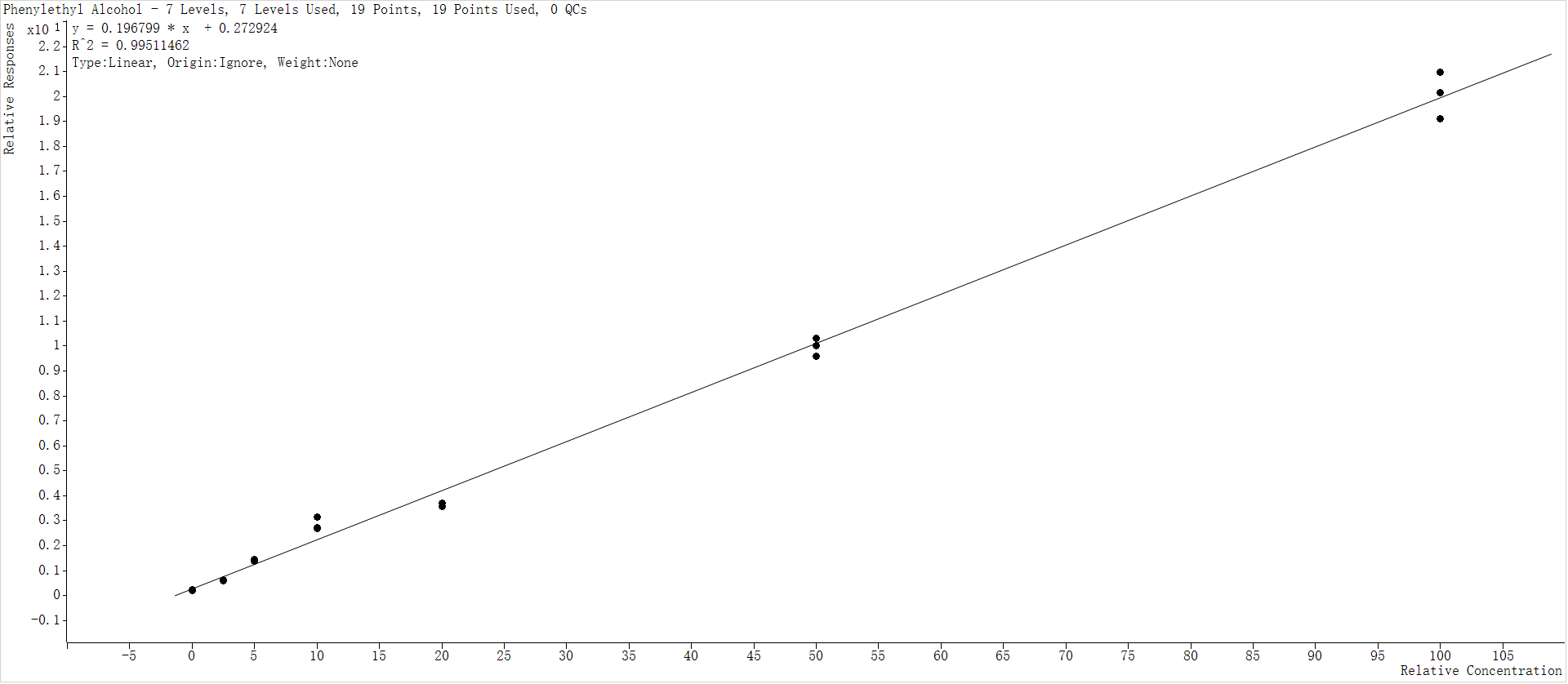
Phenethyl alcohol (for haploids)


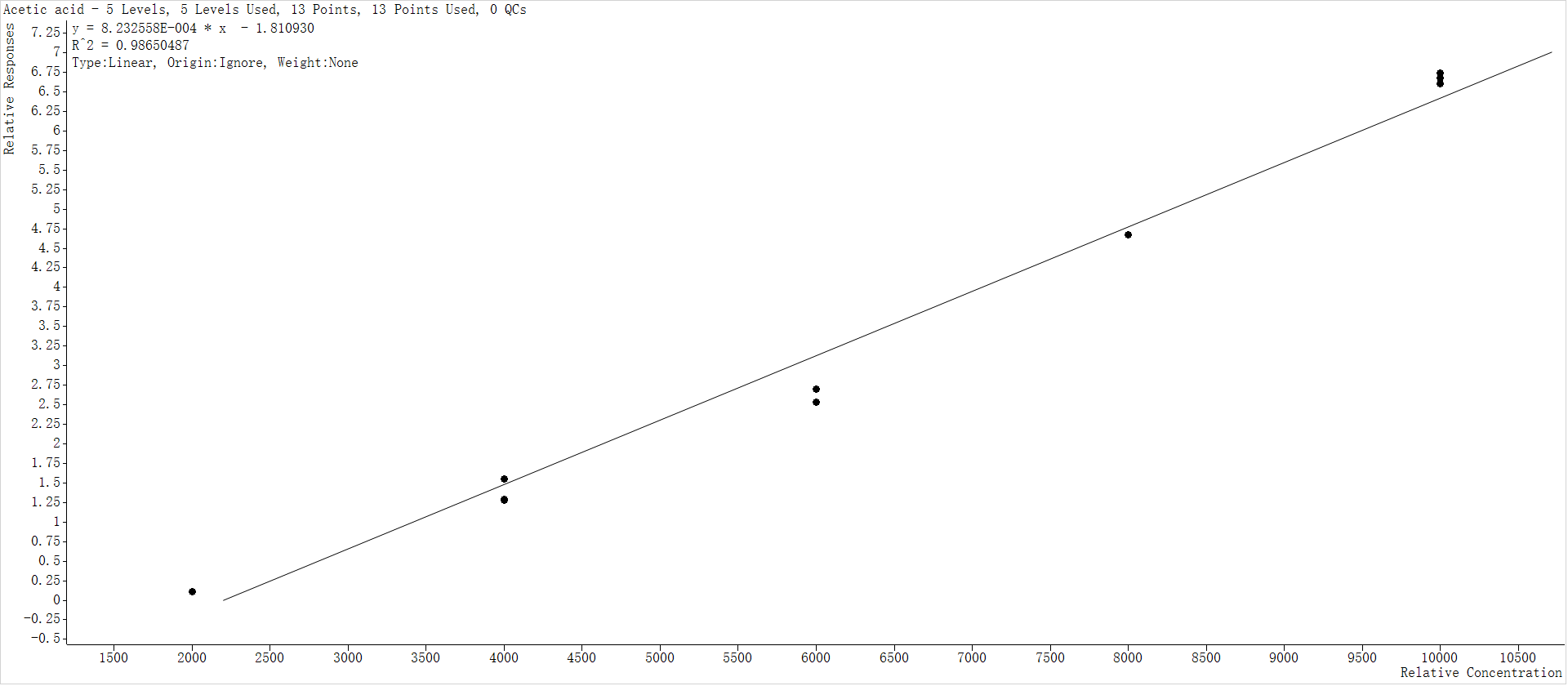
Acetic acid (for diploids)


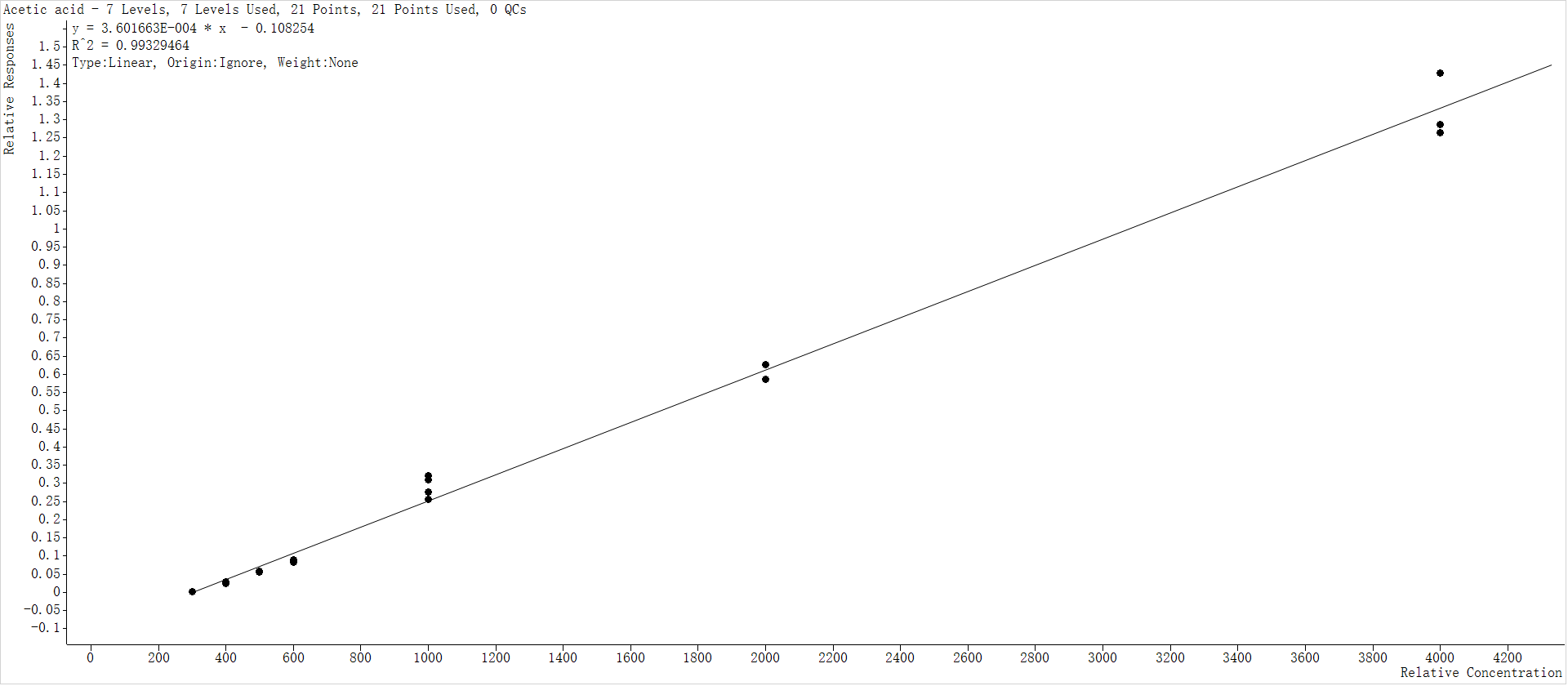


Acetic acid (for haploids)


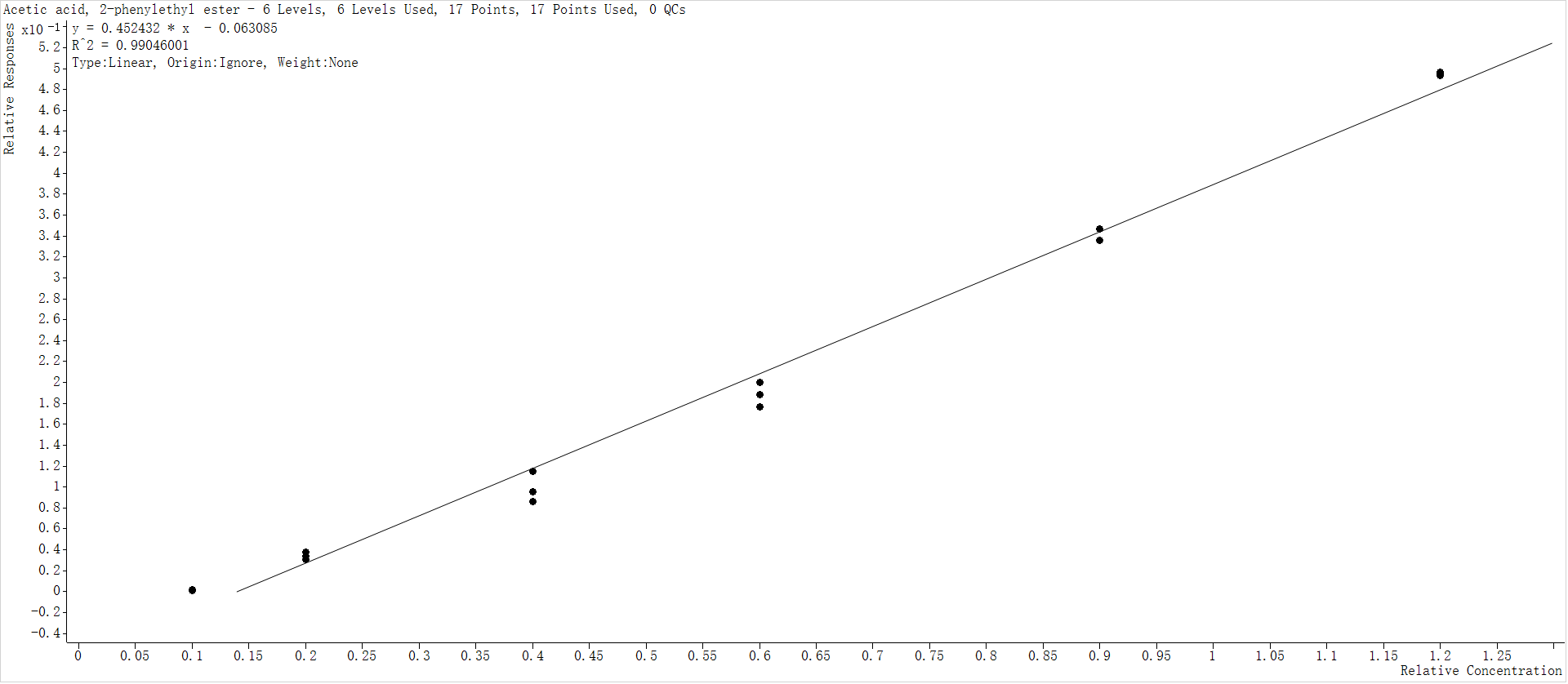


2-Phenethyl acetate


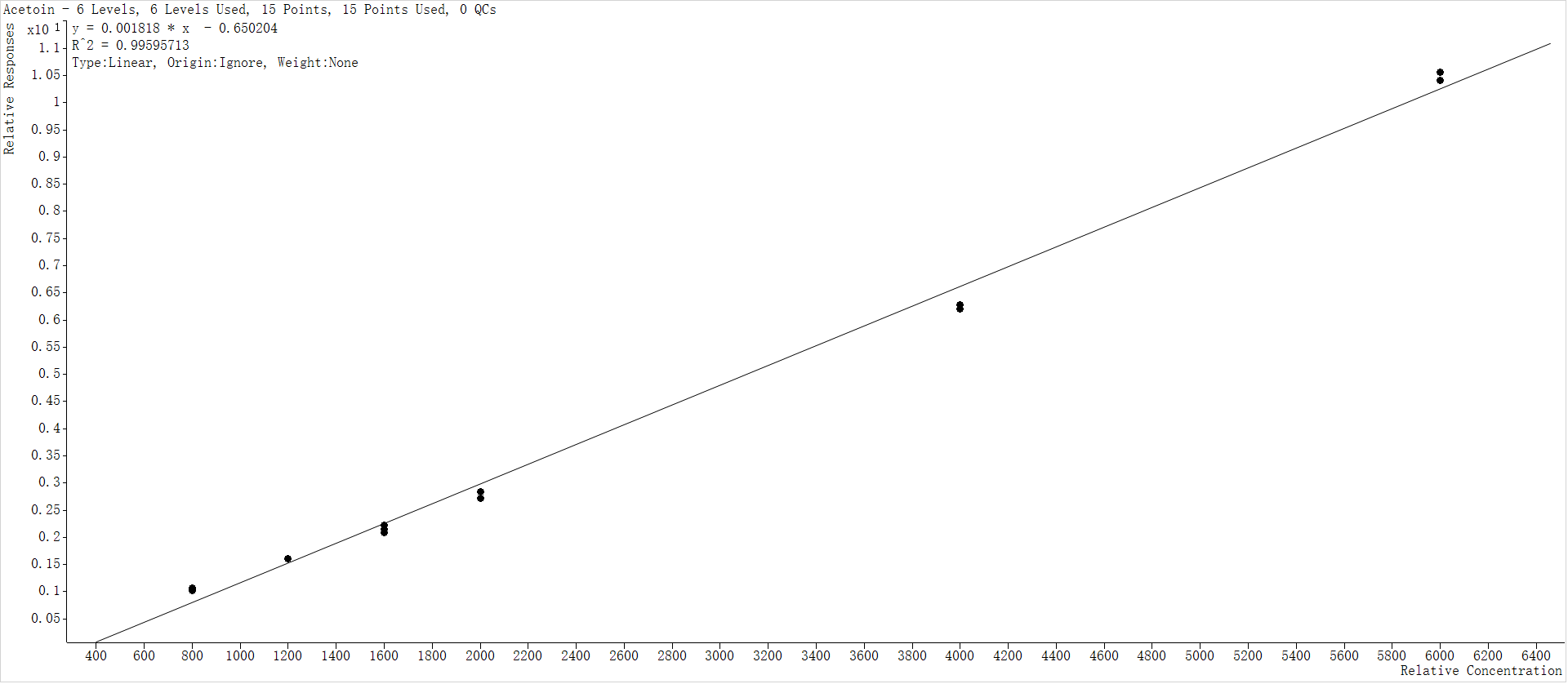


Acetoin (for diploids)


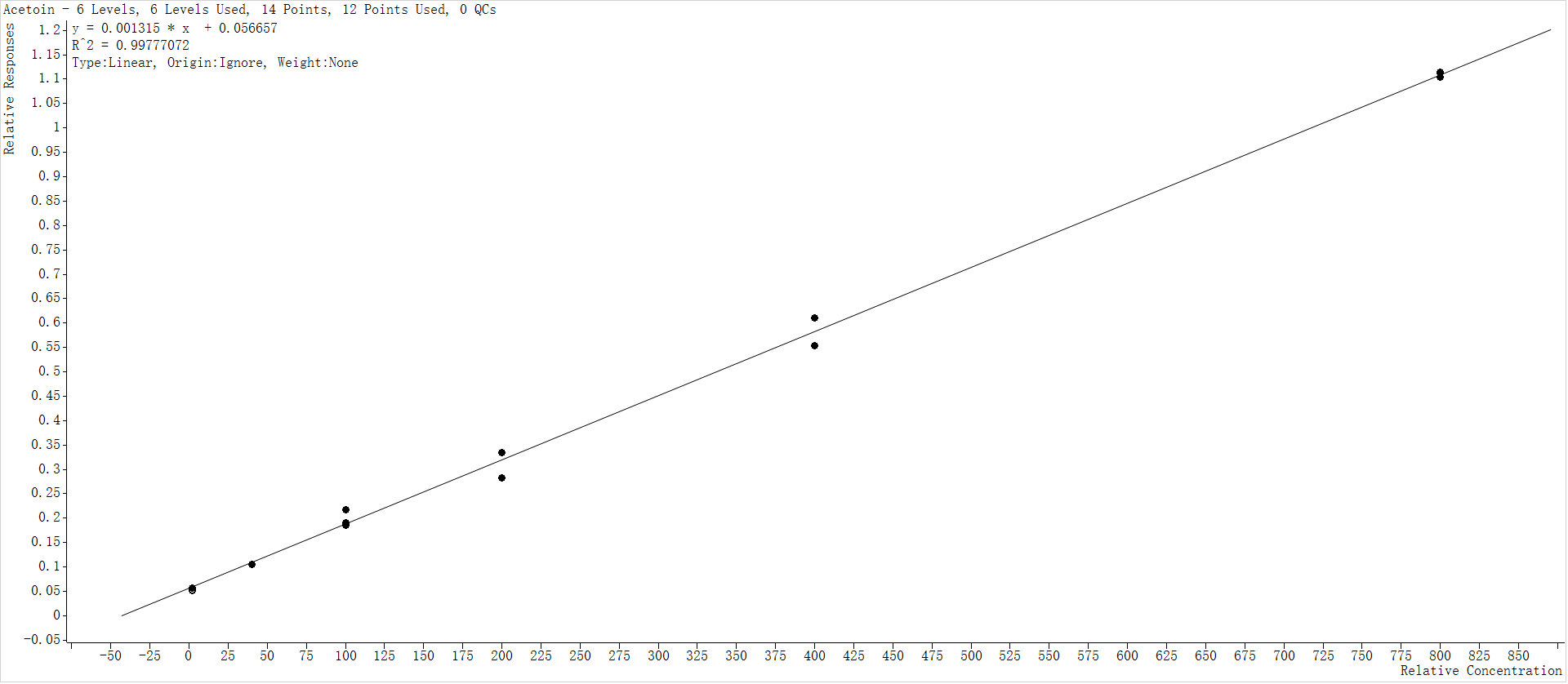


Acetoin (for haploids)


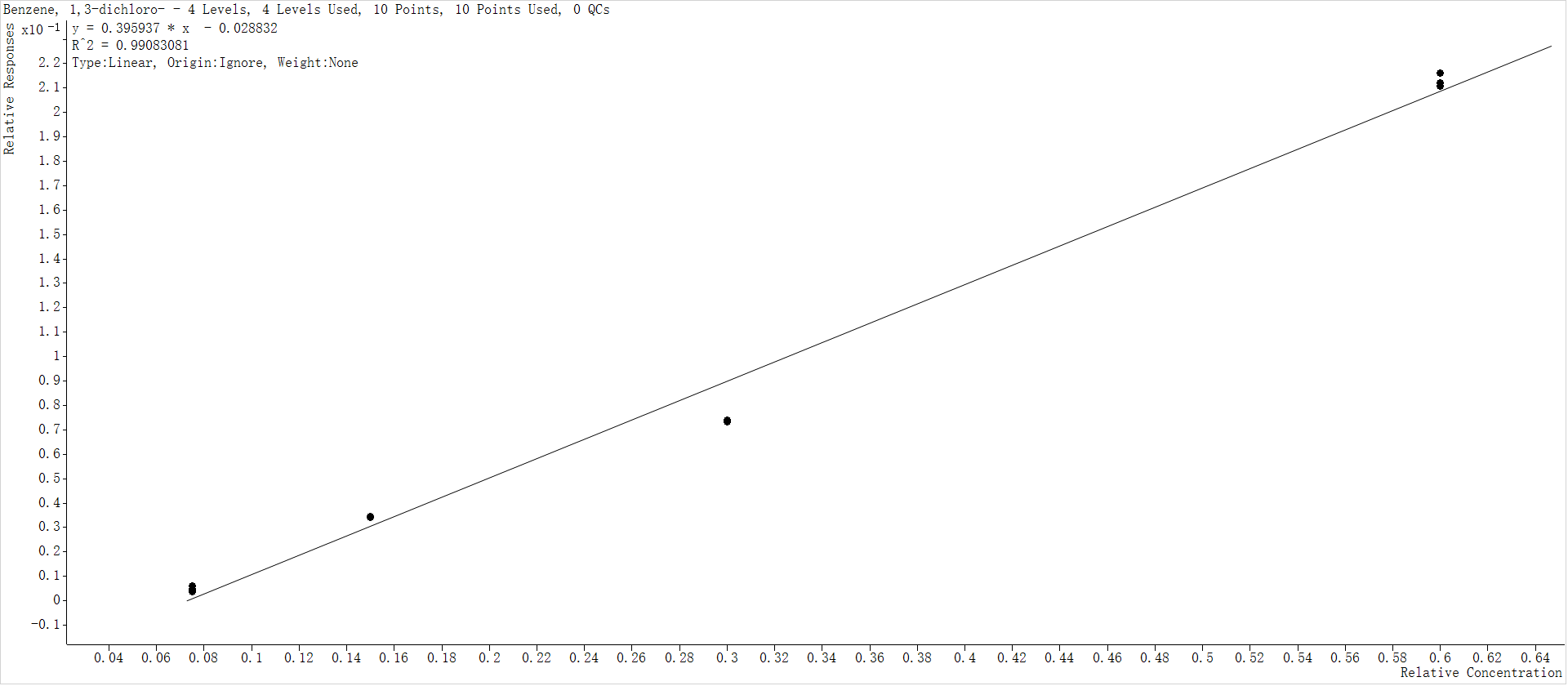


1,3-Dichlorobenzene


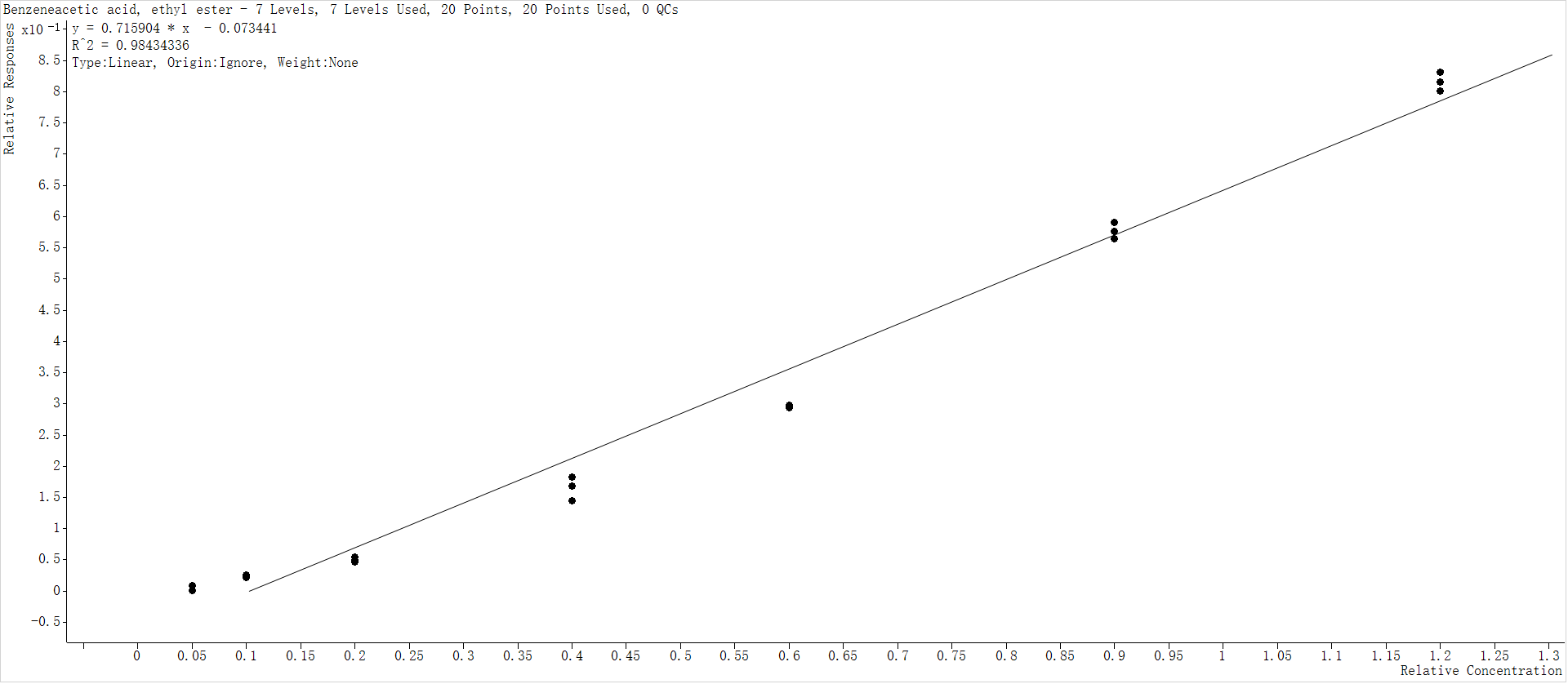


Ethyl phenylacetate


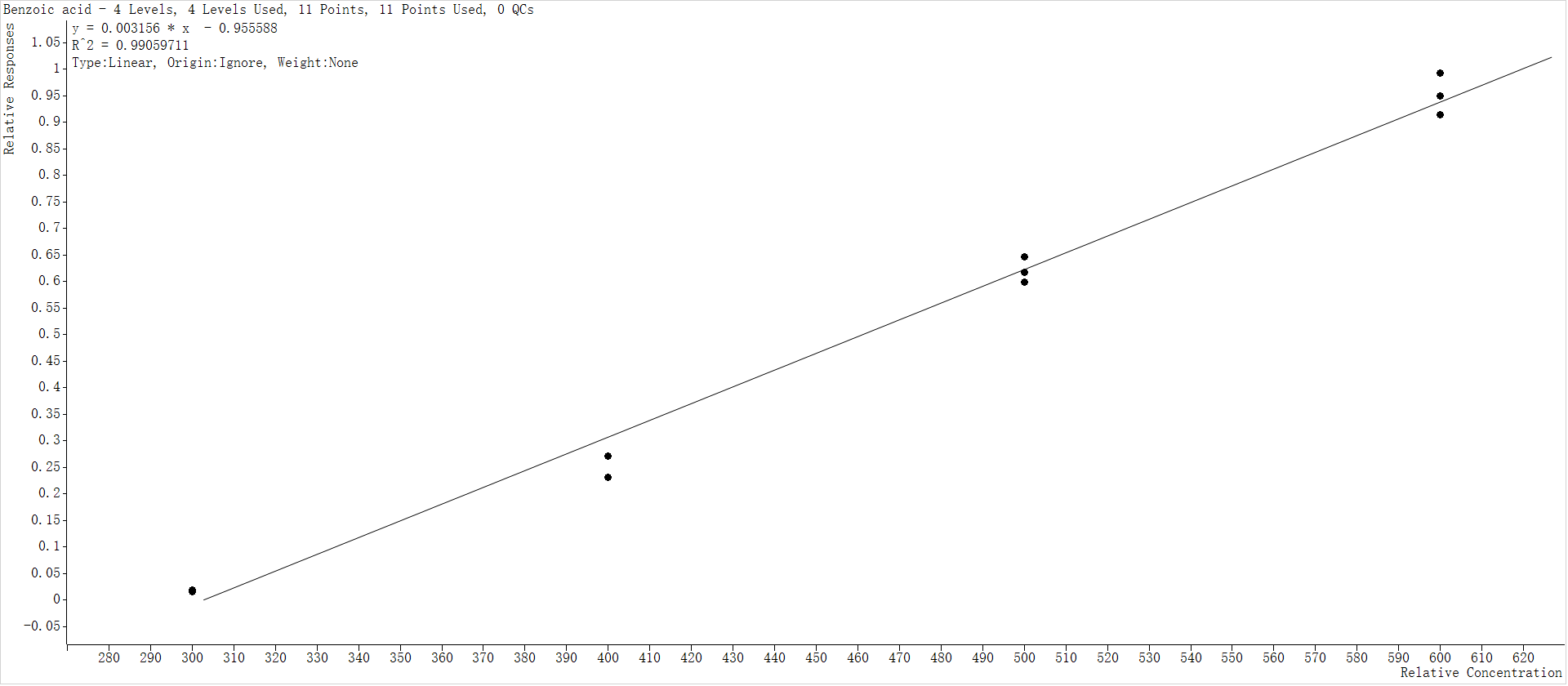


Benzoic acid


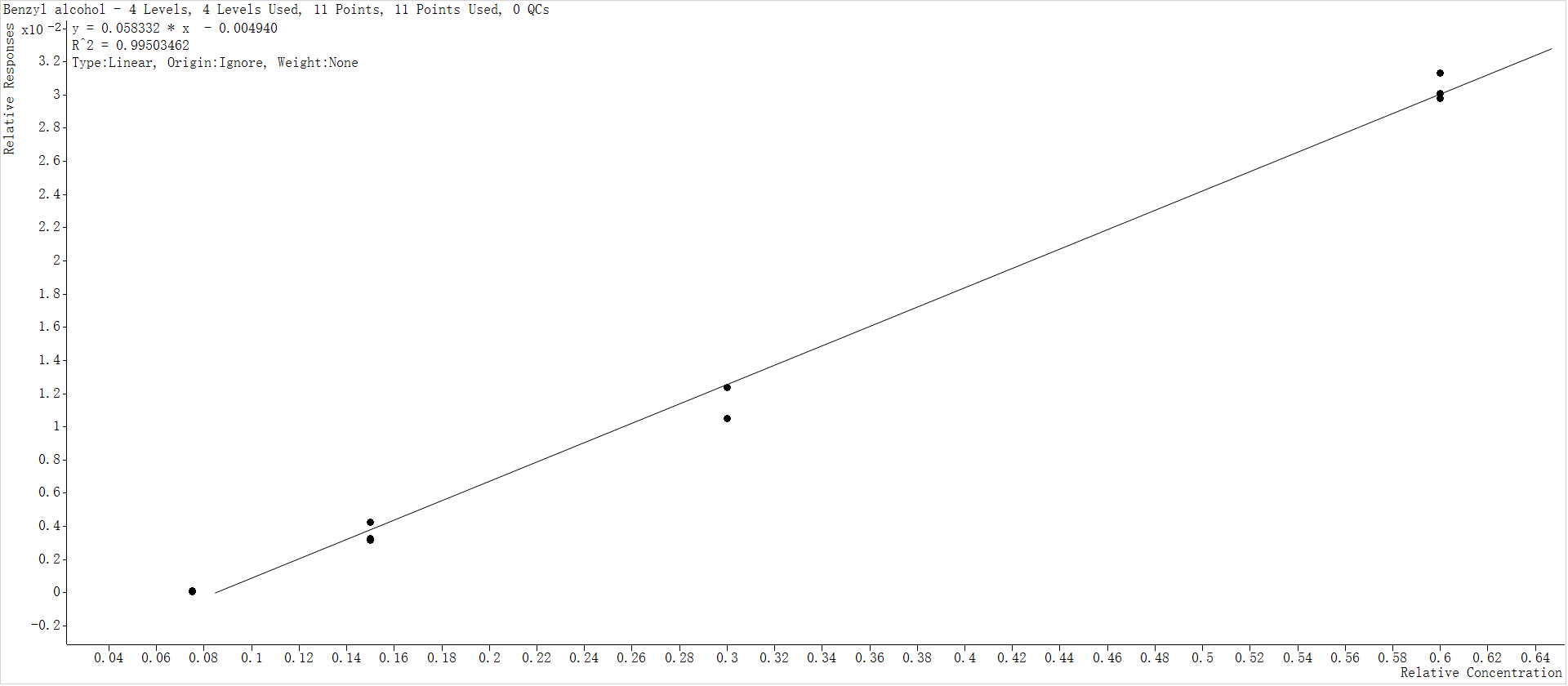
Benzyl alcohol


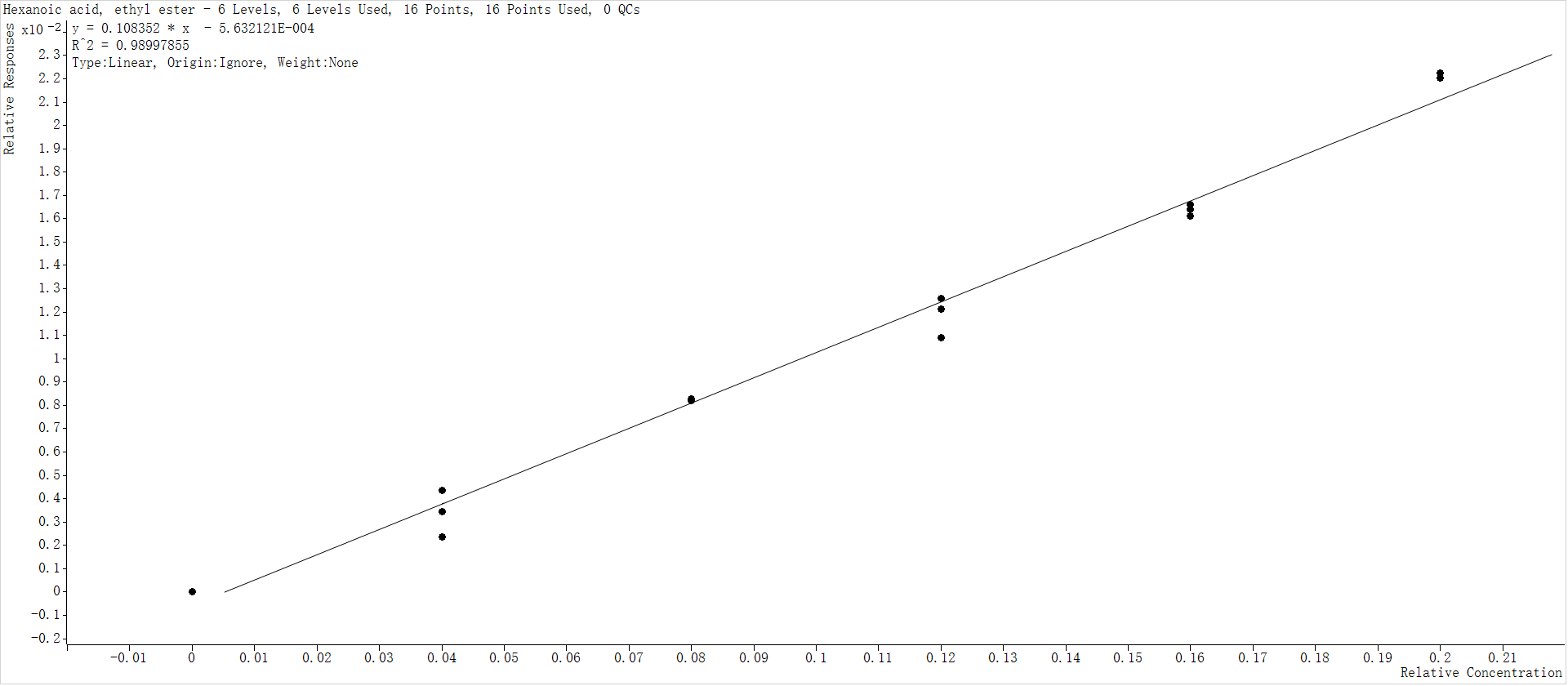
Ethyl hexanoate


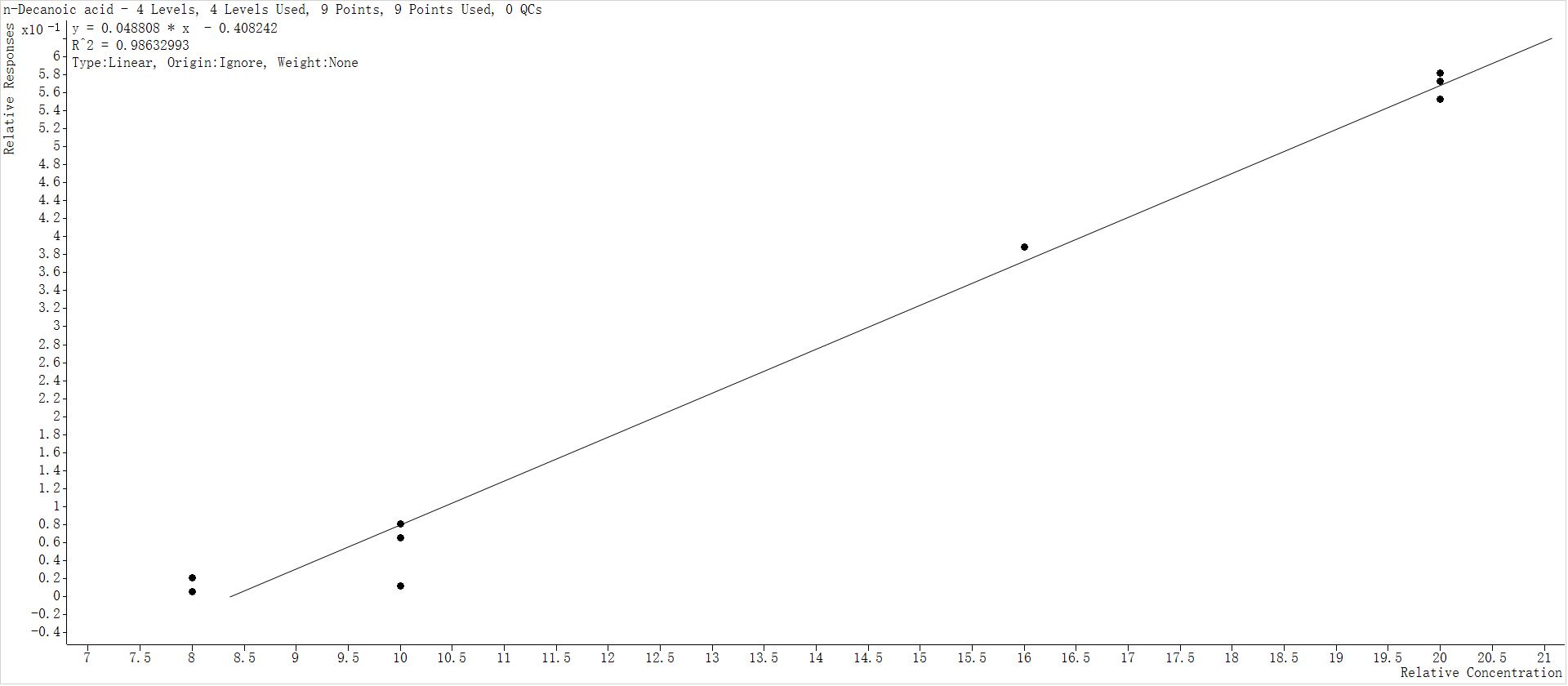
Decanoic acid


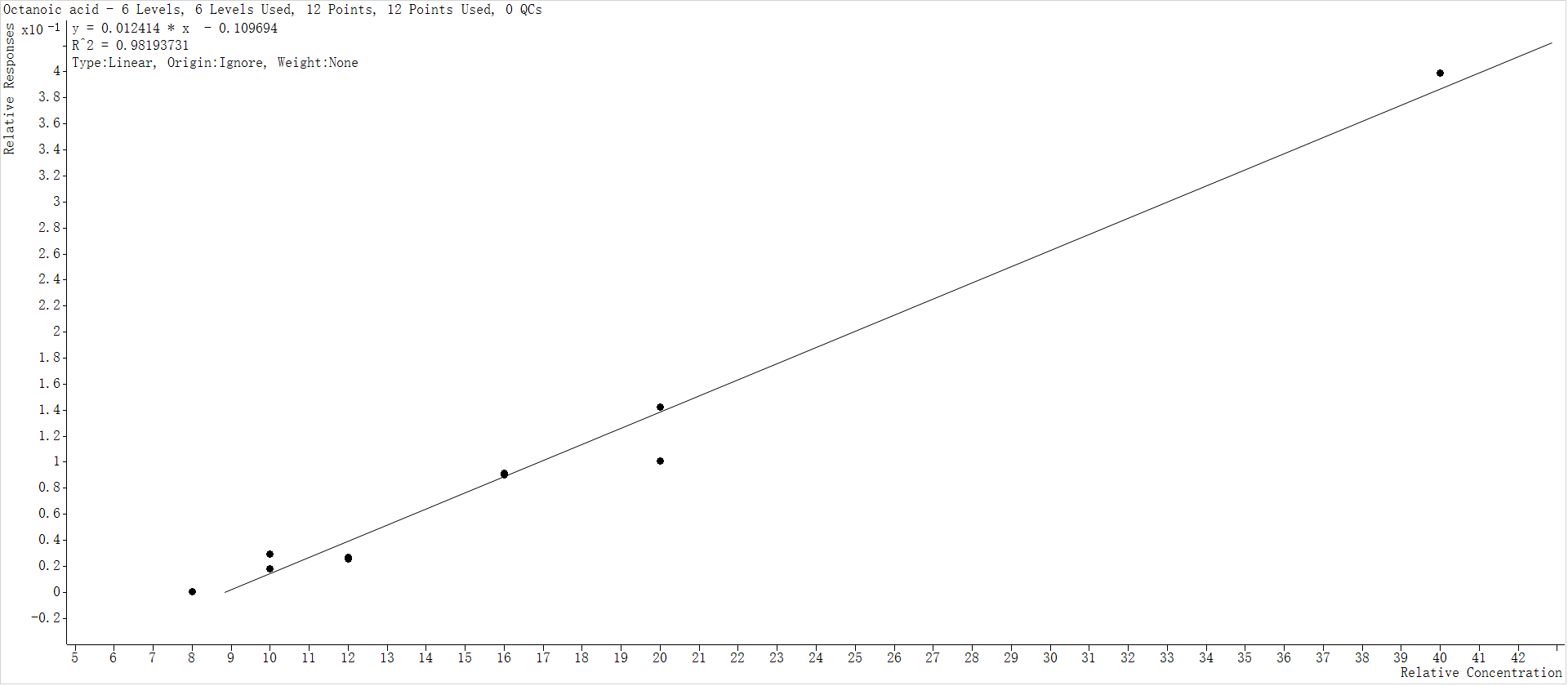
Octanoic acid


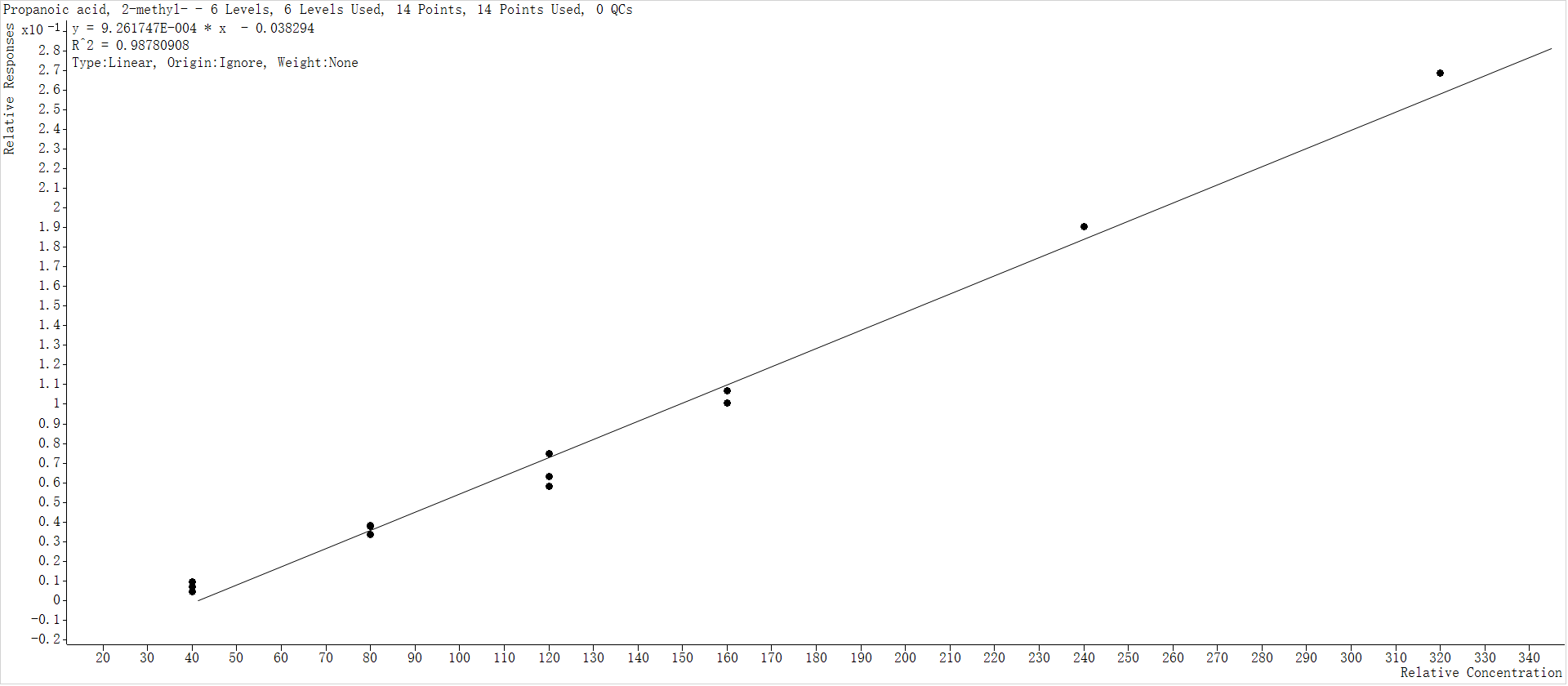
Isobutyric acid

Fig S5.


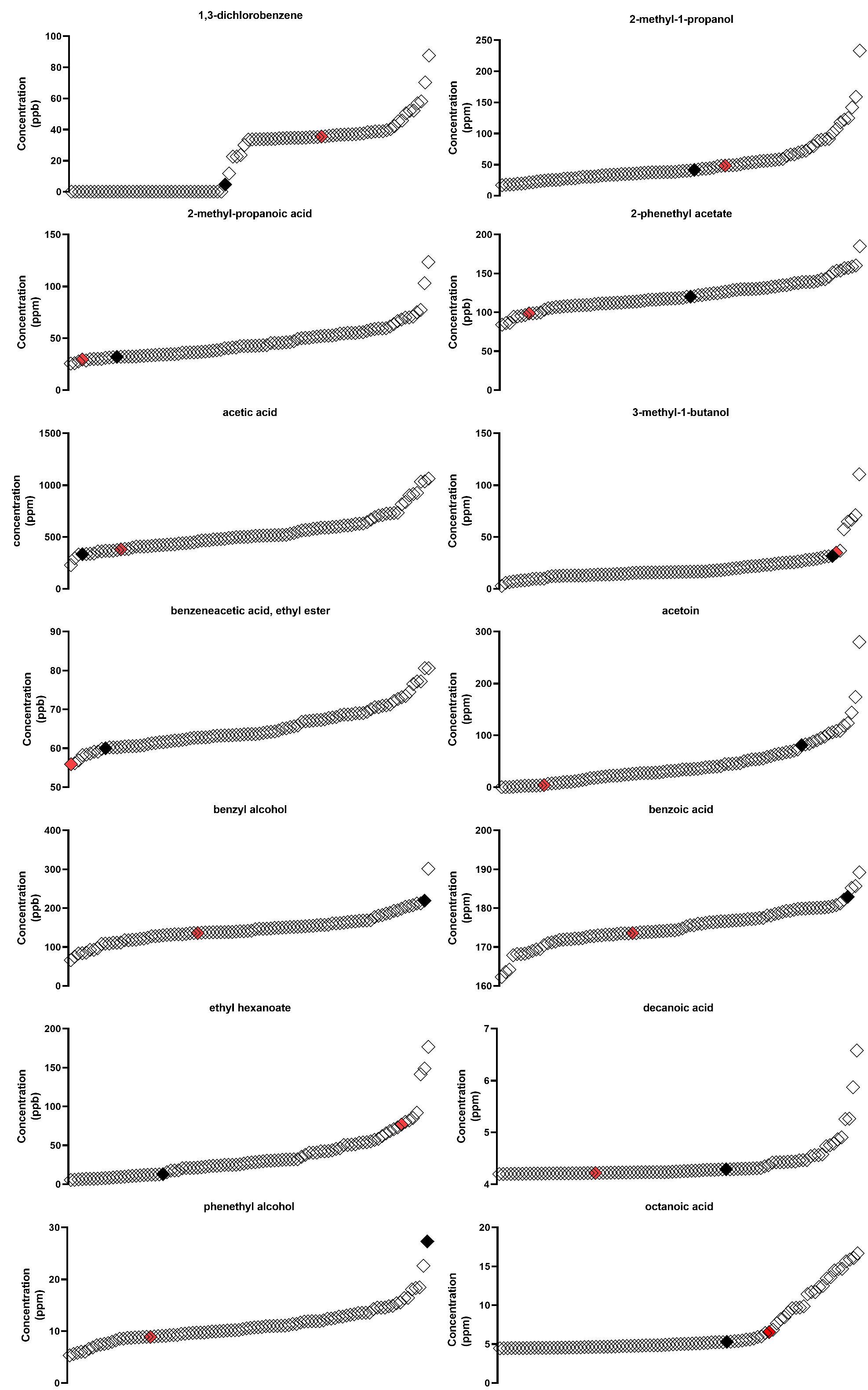


Fig S6.


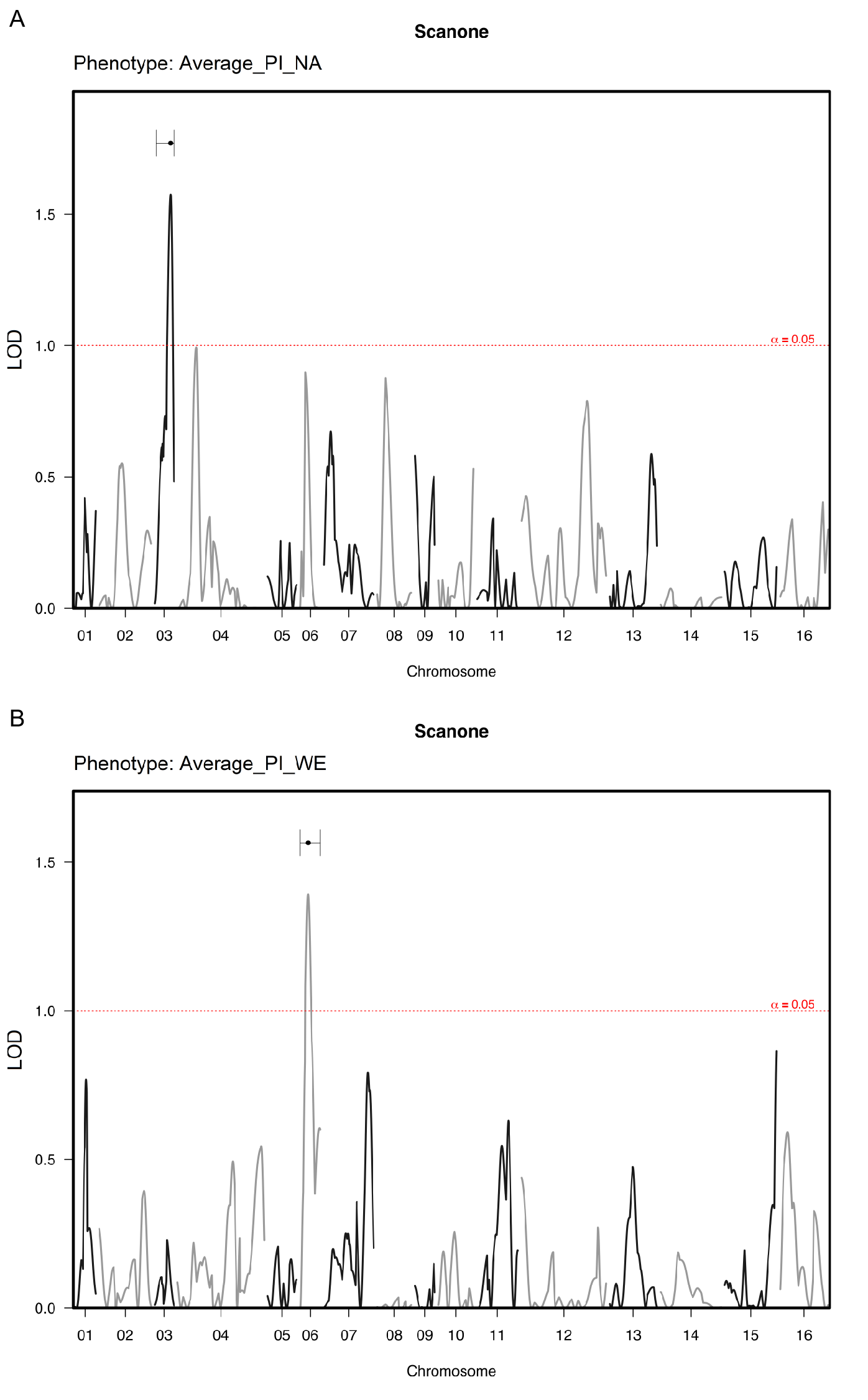


Fig S7.


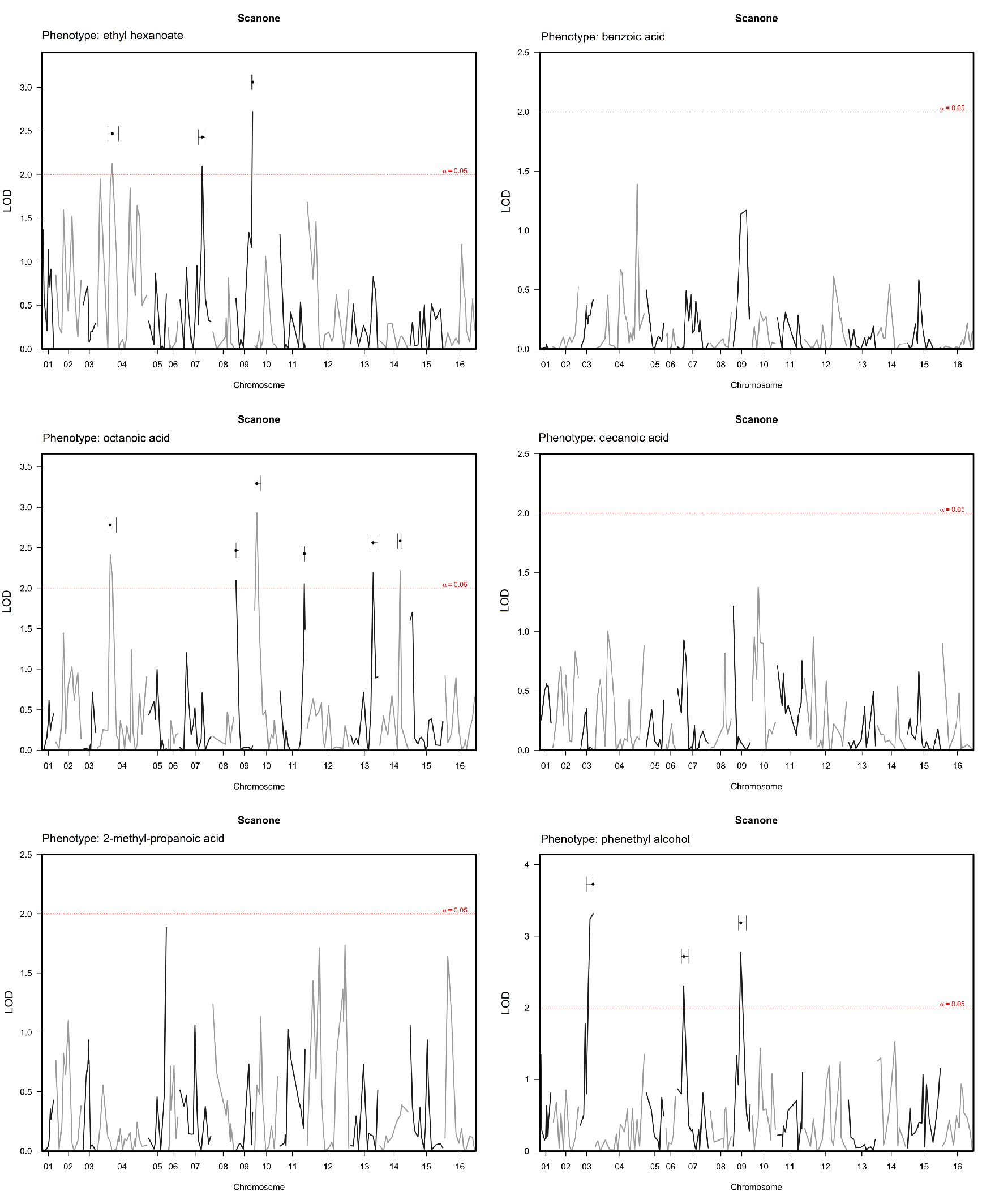


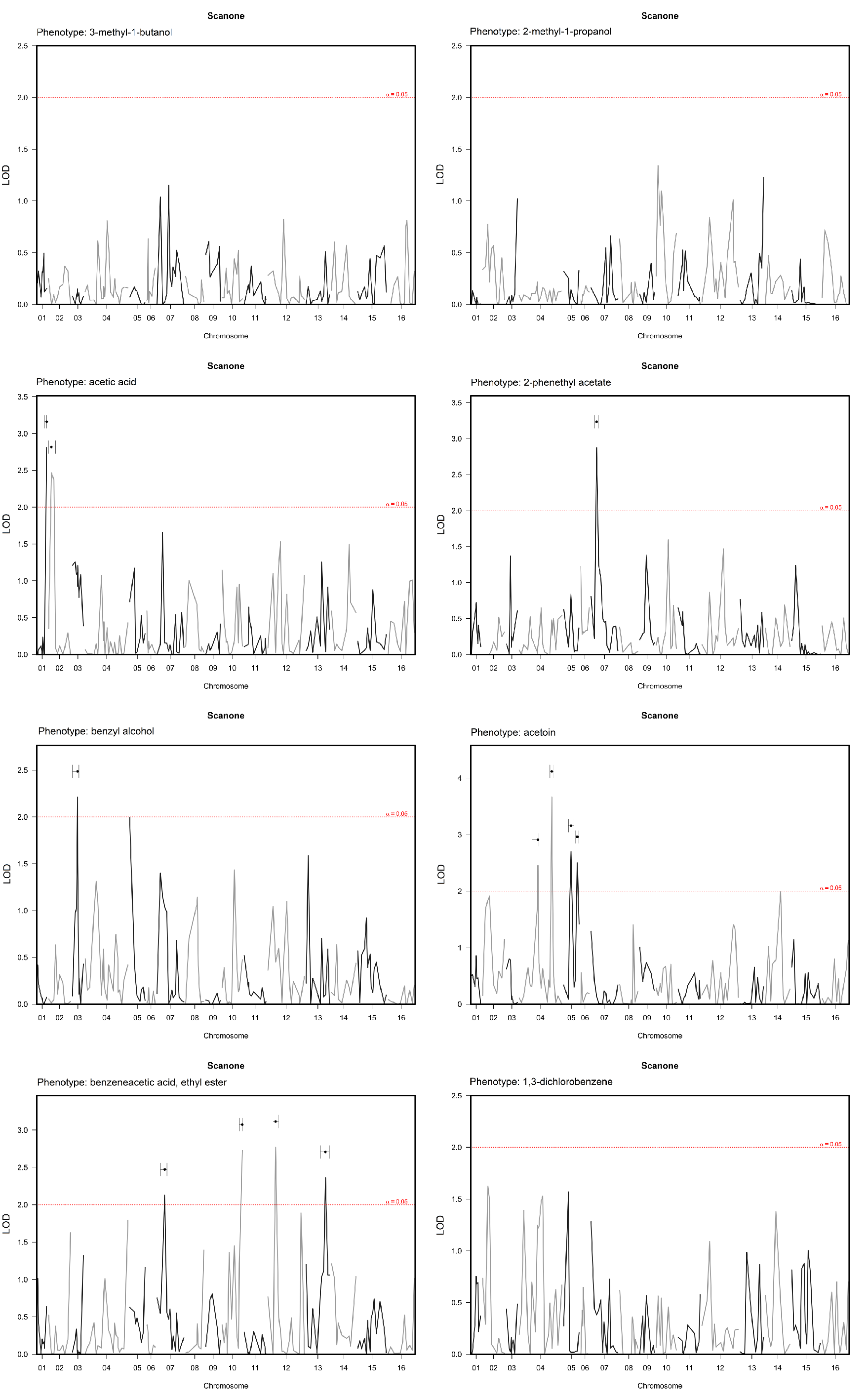


Fig S8.


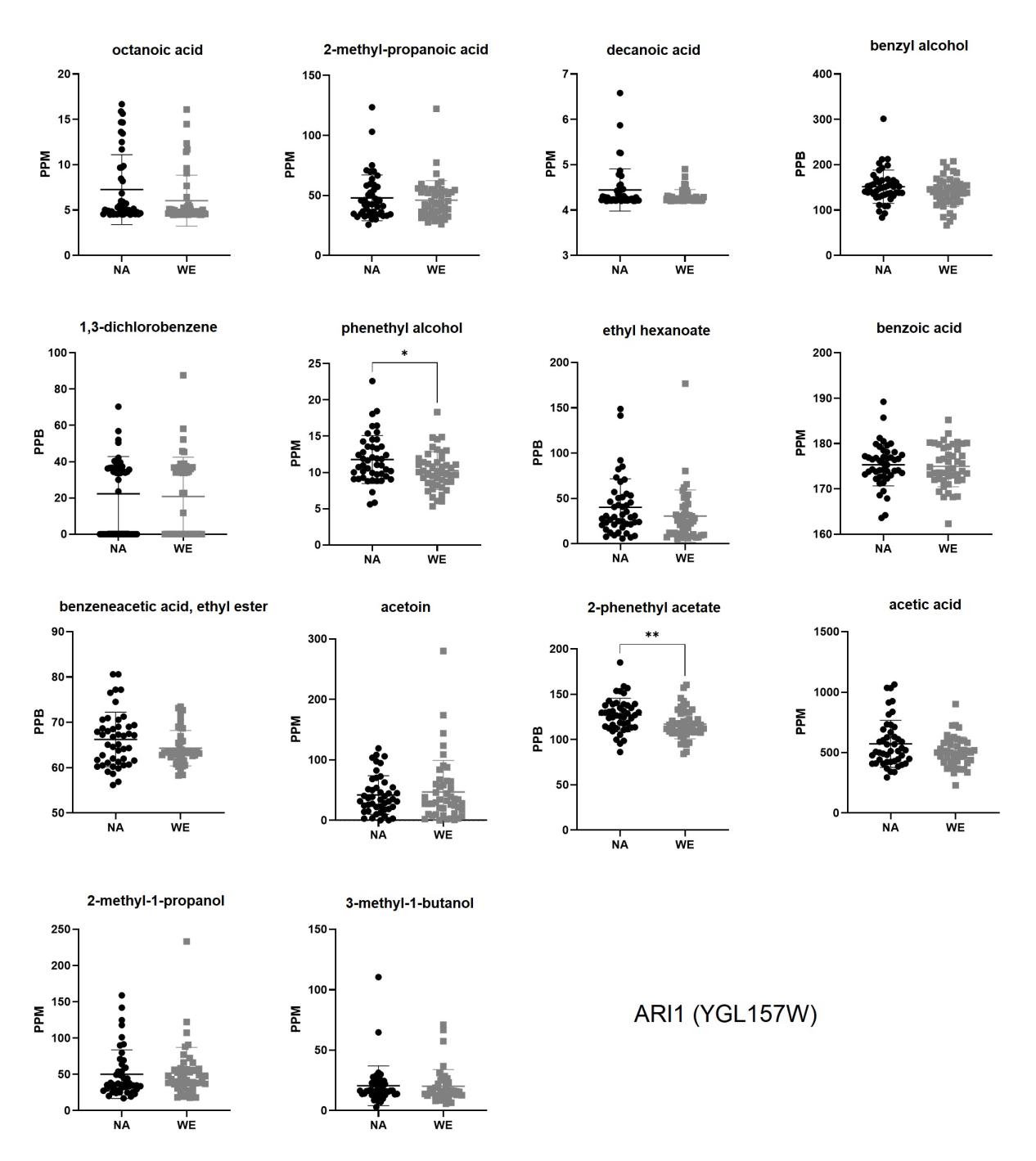

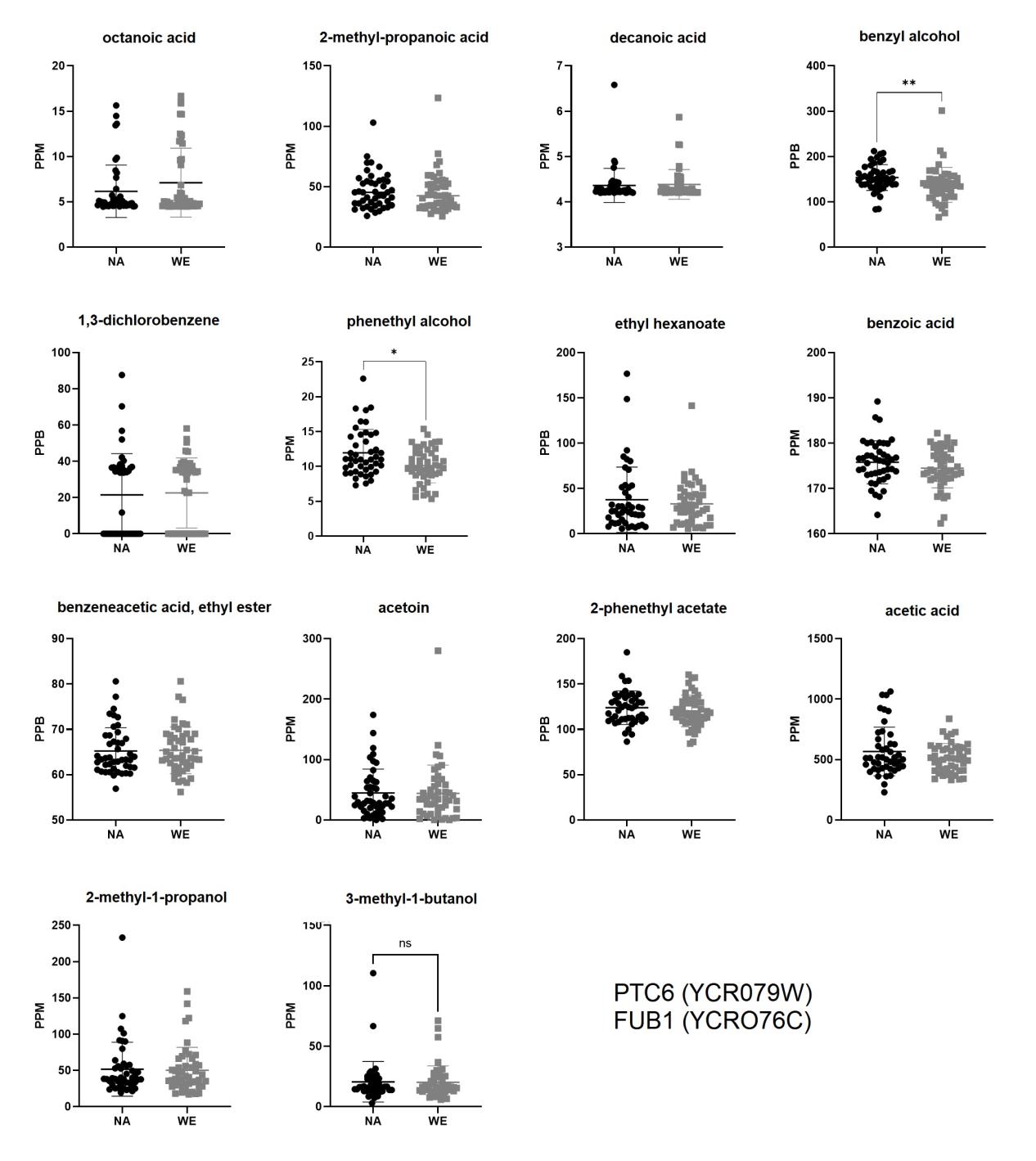

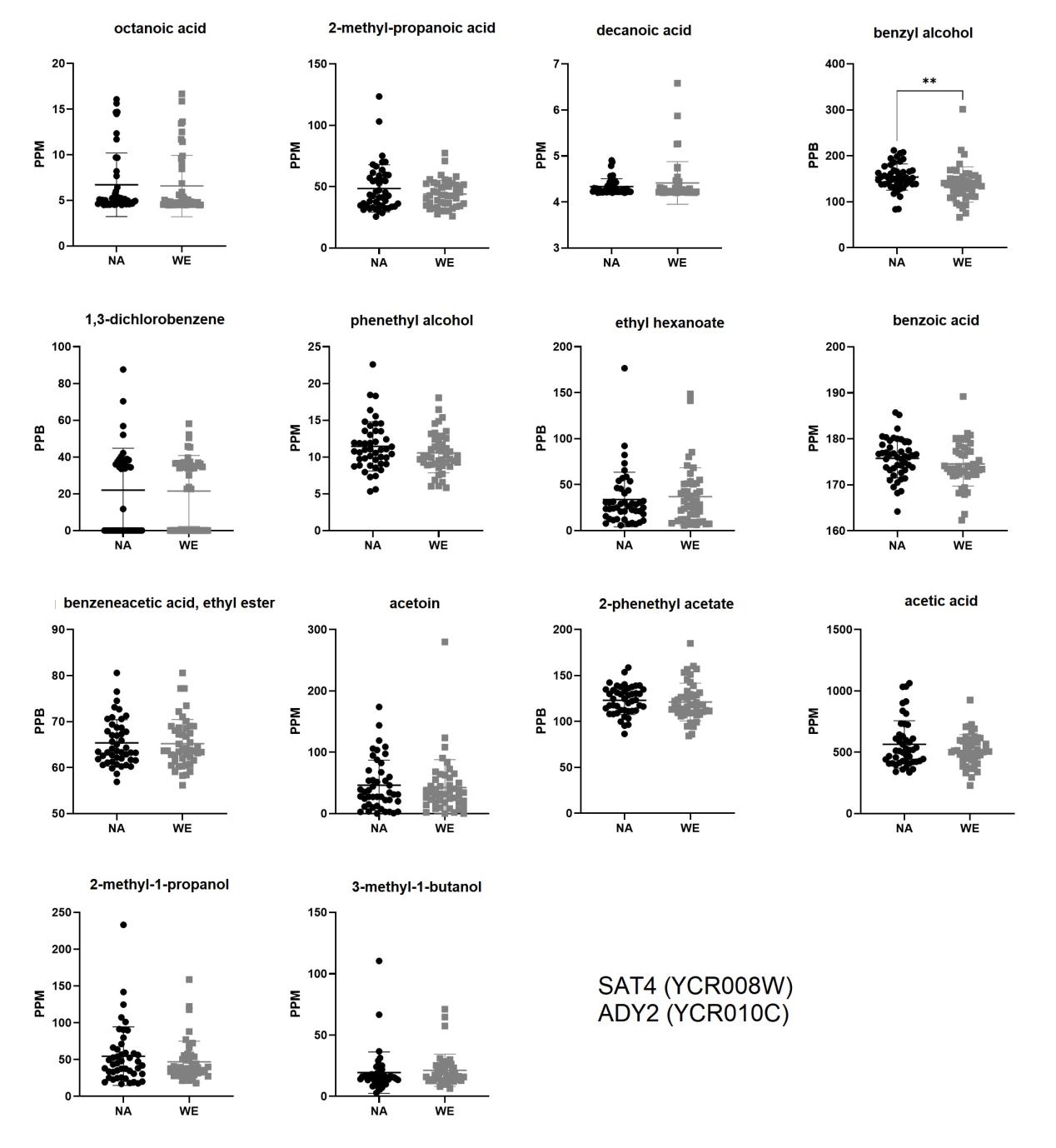


Fig S9.


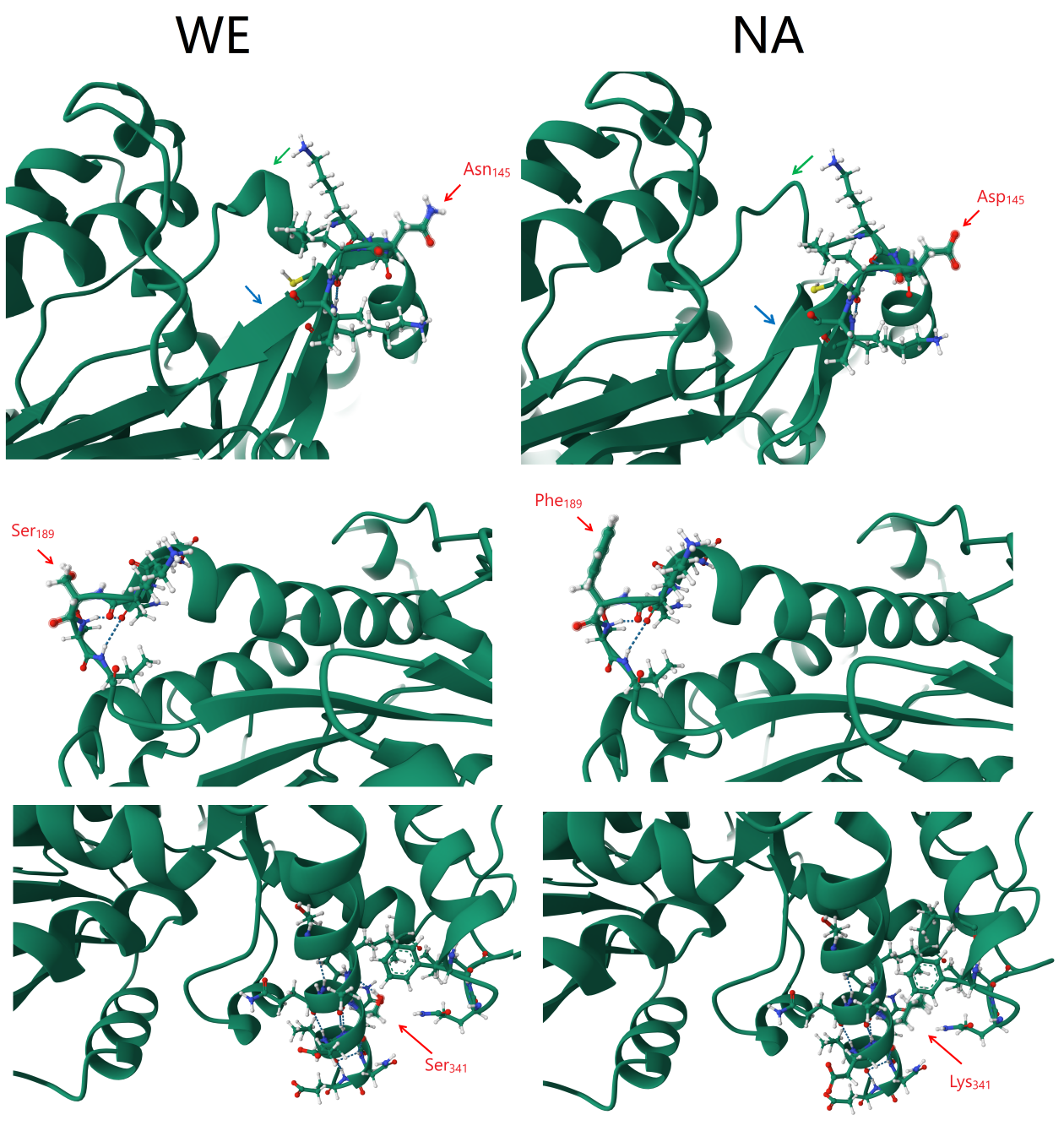

Supplement: iyae048_Supplementary_Data [file iyae048_supplementary_data.zip › Supplemental_Figures_GENETICS-2024-306838.docx]
