# Supplementary material for: Quantitative genetic analysis of attractiveness of yeast products to Drosophila
Source: Genetics. 2024 Apr 1;227(2):iyae048. doi: 10.1093/genetics/iyae048 (PMC11151935; doi:10.1093/genetics/iyae048)
Supplement: iyae048_Supplementary_Data [file iyae048_supplementary_data.zip › Supplemental_Material_Legends_GENETICS-2024-306838.docx]

## Supporting information legends

Fig S1. The apparatus used for preference assay and initial evaluations. A: front view; B: side view; C: back view; D: the trap box. The T-maze has several components: loading tubes, slide door, main body, glass corridor, and trap box. The flies are loaded into the loading tubes without CO_2_ anesthesia. The loading process is performed using a specific designed blow tube. The test yeast fermentation samples are loaded into the orange cup inside the trap boxes. At the start of the experiment, the slide door is lifted, allowing all flies to enter the main body, which further let the flies make choices via the glass corridor. The transparent back of each channel is designed for other experiments that might require photoattraction.

Fig S2. Preliminary tests for determining the feasibility of the T-maze. Eight independent preference tests were performed for preference (water, water, food, food, etc.), and each experiment was performed twice (food represents the cornmeal food for fly maintenance; grape represents diluted grape juice). The left-hand side shows the results of the one-sample t-test, revealing significant differences between the preference for cornmeal fly food and water (* P < 0.05). On the right-hand side are the results of t-tests between the two replicates for each condition; none are significant.

Fig S3 A. Residual sugar of fermentates from six yeast strains. Sugar was measured by refractometer after fermentation. Each bar represents the mean of three replicates and error bar stands for standard deviation. One-way ANOVA is used for testing the significance among all samples (* P < 0.05).

Fig S3 B. Alcohol yield of fermentates from six yeast strains. Alcohol concentration was measured by vinometer after fermentation. Each bar represents the mean of three replicates and error bar stands for standard deviation. One-way ANOVA is used for testing the significance among all samples (* P < 0.05).

Fig S4. Fourteen compounds were quantified using calibration lines plotted using analytical standards. The concentration scales for the calibration lines were determined from the approximate concentrations of the samples.

Fig S5. Phenotypic distribution of F1 progeny. Each box represents the mean concentration of one segregant (n = 3). The colored boxes represent the mean concentration of the parental strains (n = 3) (red box: WE; black box: NA). All individual values are sorted from minimum to maximum.

Fig S6. QTL plots were generated from two sets of preference indices when segregants challenged the parental strains. A (against NA) and B (against WE) represent the whole-genome scan of the two traits. The threshold LOD value was the horizontal line (α = 0.05).

Fig S7. QTL plots generated from the concentrations of 14 quantified compounds. The QTL analysis was based on 92 verified F1 segregants. Images represent the whole genome scan-one output (standard analysis) of 14 traits. The significance threshold (α = 0.05) of the LOD value is indicated by a horizontal line.

Fig S8. The quantified concentrations of Individual segregant categorized by alleles. The F1 progeny was categorized into two groups depending on their candidate alleles. The comparison of concentrations measured using GC-MS between the groups was designed to identify the allelic impact on the distribution of traits. *P < 0.05, **P < 0.01

Fig S9. Prediction of protein structure encoded by *ARI1* alleles using ALHPAFOLD. The protein encoded by two alleles differed in three amino acids, namely, 145 (upper panel), 189 (middle), and 341 (lower); left WE, right NA. Red arrows, positions of different amino acids; green, missing beta-fold, blue; putative variation of beta-fold; black, potential structure alteration of protein binding site.

Table S1. List of volatile compounds quantified using GC-MS profiling after yeast fermentation.

Table S2. Amino acid alignment of candidate genes

Table S3. Primers used in this study

Table S4. Strains used in this study

Table S5. Strains for reciprocal hemizygosity assay

Table S6. Thermocycles for Polymerase Chain Reactions
