# Supplementary material for: Quantitative genetic analysis of attractiveness of yeast products to Drosophila
Source: Genetics. 2024 Apr 1;227(2):iyae048. doi: 10.1093/genetics/iyae048 (PMC11151935; doi:10.1093/genetics/iyae048)
Supplement: iyae048_Supplementary_Data [file iyae048_supplementary_data.zip › Supplemental_Tables_GENETICS-2024-306838.docx]

## Supplementary data tables

Table S1.

| Aroma compounds | CAS number | Formula | Molecular weight | Retention time | Chemical group |
| --- | --- | --- | --- | --- | --- |
| Ethyl phenylacetate | 101-97-3 | C_10_H_12_O_2_ | 164.084 | 36.5379 | Ester |
| 2-Phenethyl acetate | 103-45-7 | C_10_H_12_O_2_ | 164.084 | 37.6102 | Ester |
| Benzyl alcohol | 100-51-6 | C_7_H_8_O | 108.058 | 40.6527 | Alcohol |
| Phenethyl alcohol | 60-12-8 | C_8_H_10_O | 122.073 | 42.2654 | Alcohol |
| Octanoic acid | 124-07-2 | C_8_H_16_O_2_ | 144.115 | 49.3837 | Acid |
| Decanoic acid | 334-48-5 | C_10_H_20_O_2_ | 172.146 | 56.2959 | Acid |
| Benzoic acid | 65-85-0 | C_7_H_6_O_2_ | 122.037 | 60.0143 | Acid |
| Isobutanol | 78-83-1 | C_4_H_10_O | 74.073 | 11.3835 | Alcohol |
| Ethyl hexanoate | 123-66-0 | C_8_H_16_O_2_ | 144.115 | 14.9763 | Ester |
| Isoamyl alcohol | 123-51-3 | C_5_H_12_O | 88.089 | 15.8959 | Alcohol |
| Acetic acid | 64-19-7 | C_2_H_4_O_2_ | 60.021 | 23.2873 | Acid |
| Isobutyric acid | 79-31-2 | C_4_H_8_O_2_ | 88.052 | 27.5399 | Acid |
| 1,3-Dichlorobenzene | 541-73-1 | C_6_H_4_Cl_2_ | 145.969 | 21.3022 | halogenated aromatic hydrocarbon |
| acetoin | 513-86-0 | C_4_H_8_O_2_ | 88.052 | 18.4507 | Ketone |

Table S2.

| Allele | Amino acid variation position | Amino acid | | |
| --- | --- | --- | --- | --- |
|  |  | WE | NA | S288C |
| *GAT1* | 103aa | Pro | Ala | Pro |
|  | 238aa | Ile | Thr | Thr |
| *BST1* | 172aa | Ser | Ser | Arg |
|  | 202aa | Thr | Ala | Ala |
|  | 253aa | Pro | Ala | Pro |
|  | 506aa | Leu | Gln | Gln |
|  | 516aa | Gly | Gly | Ala |
|  | 610aa | Arg | Lys | Lys |
|  | 823aa | Leu | Val | Leu |
|  | 824aa | Val | Ile | Val |
|  | 849aa | Val | Asp | Asp |
|  | 908aa | Ser | Ser | Asn |
| *YFL040W* | 192aa | Ile | Met | Ile |
| *FUB1* | 18aa | Cys | Gly | Gly |
|  | 50aa | Pro | Ser | Ser |
|  | 169aa | Arg | Arg | Gly |
|  | 183aa | Arg | Arg | Gly |
| *PTC6* | 92aa | Ser | Pro | Pro |
|  | 103aa | Arg | Lys | Lys |
| *ARI1* | 21aa | Val | Val | Met |
|  | 145aa | Asn | Asp | Asp |
|  | 189aa | Ser | Phe | Ser |
|  | 341aa | Ser | Leu | Leu |
| *SAT4* | 243aa | Thr | Ser | Thr |
|  | 281aa | Cys | Gly | Cys |
|  | 600aa | Arg | His | Arg |

Table S3.

| Name | Sequence(5′-3′) | Note |
| --- | --- | --- |
| *FUB1*_FW | TCCTACCCTTTGTAGATCATTTATTGTATA | Specific deleting primers |
| *FUB1*_RV | CACGGTAACGTTATAGAGTCTACCGG |  |
| *PTC6*_FW | GTACATGTTTCTTTTCATTCACCTTGTTTC |  |
| *PTC6*_RV | TTCGATTAGCATCAAAAATCTCAGC |  |
| *YFL040W*_FW | CGTTGTGACGCTCAGCCAAGA |  |
| *YFL040W*_RV | TGTGCGCGTATGTTTATGTATGTACCTC |  |
| *BST1*_FW | AAATGAGTCTCAACACATTCCTTGCA |  |
| *BST1*_RV | AGTAGTGGTAGAAACCACCTTTGCTTT |  |
| *GAT1*_FW | GGTGCAGCTACCGCTGGTATTAACAG |  |
| *GAT1*_RV | GCGCCTTAGCTTTTGATAAATGTTGCA |  |
| *SAT4*_FW | AGTTGTACTAAGCCTTTAAGCGT |  |
| *SAT4*_RV | CGAATGCCGGATAACAAAGCA |  |
| *ADY2*_FW | CTATGGCGCCTTCTCCTCTG |  |
| *ADY2*_RV | AGTGTACGACTGAAACAGCCA |  |
| *ARI1*_FW | GCATAGGATTTTCCGCGGTTC |  |
| *ARI1*_RV | GCGCTATATGAATTCGTGGCA |  |
| *FUB1*_A | CAAGATTTGAATTTGTTCCTAAACG | Deletion validating primers |
| *FUB1*_B | AGGTAAGCTTTCTCTTTCTAGTGGC |  |
| *FUB1*_D | CAATCTGATATTAAGGGAACCATTG |  |
| *PTC6*_A | GCTATTTTCTAACCCAAAGAAGGAC |  |
| *PTC6*_B | CTTGCATATTCCTGTACCAACTTCT |  |
| *PTC6*_D | CACAAAGTTTGCTAGGATTTGATCT |  |
| *YFL040W*_A | CTGAAAATGAAATAGAGGTTTCTCG |  |
| *YFL040W*_B | GCGTAGGATGCTACTAAAATTGAAA |  |
| *YFL040W*_D | TATCGTTCACCACAAGTGTTTCTAA |  |
| *BST1*_A | GCATAACTCACTAGGTACCCTCAAA |  |
| *BST1*_B | TAGAGAAAAGGCGATATGTGTAAGG |  |
| *BST1*_D | CAAAATTTACGGCTTTGAAAAAGTA |  |
| *GAT1*_A | GGCTGATAAGGGAGAAGATAAGATAA |  |
| *GAT1*_B | ACACATTTTCAAAAACAGAAGATCC |  |
| *GAT1*_D | GGTTGCTCTTACTGAGCTTCTAGTG |  |
| *SAT4*_A | AGACCTTTACTTGGAGTTTCGATTT |  |
| *SAT4*_B | TGCCTCACCACGTAATTTGC |  |
| *SAT4*_D | ATAATAACCGCGGGCATGTG |  |
| *ADY2*_A | AGACTGCATTTTCTTACAGCTTTTT |  |
| *ADY2*_B | TCGCATTGAACATGGACAGC |  |
| *ADY2*_D | TGTTCAAAATCCGCGCATTT |  |
| *ARI1*_A | TACGCCAGCTCTCTAGTTACAGTTT |  |
| *ARI1*_B | TTTGTACCGTTCACTGCAGG |  |
| *ARI1*_D | CCAAACCTTAACGTGATCATGAT |  |
| KanB | CTGCAGCGAGGAGCCGTAAT |  |
| KanC | TGATTTTGATGACGAGCGTAAT |  |
| MAT_P1 | AGTCACATCAAGATCGTTTATGG | Mating type primers |
| MAT_P2 | GCACGGAATATGGGACTACTTCG |  |
| MAT_P3 | ACTCCACTTCAAGTAAGAGTTTG |  |

Table S4.

| **Name of strain** | **Lab ID** | **Origin** | **Aliases** | **Genotype** | **Note** |
| --- | --- | --- | --- | --- | --- |
| Wine European (WE) | OS03 | SGRP | DBVPG6765 | Wild type | Strain information: https://www.nature.com/articles/nature07743 |
| West African (WA) | OS60 | SGRP | DBVPG6044 | Wild type |  |
| S288C | OS96 | SGRP | S288C | Wild type |  |
| North American (NA) | OS104 | SGRP | YPS128 | Wild type |  |
| Sake (SA) | OS253 | SGRP | Y12 | Wild type |  |
| YJM978 | OS303 | SGRP | YJM978 | Wild type |  |
| WE A | OS587 | SGRP | DBVPG6765 | hoΔ::HphMX MATa ura3-delta0 leu2-delta0 lys2-delta0 | Strain information： https://academic.oup.com/genetics/article/195/3/1141/5935492 |
| NA Alpha | OS605 | SGRP | YPS128 | hoΔ::HphMX MATalpha ura3-delta0 leu2-delta0 lys2-delta0 |  |
| WE × NA F1 Segregants | FS101-FS196 | SGRP | AE01FC | hoΔ::HphMX MATa (or MATalpha) ura3-delta0 leu2-delta0 lys2-delta0 | Strain information： https://onlinelibrary.wiley.com/doi/full/10.1111/j.1365-294X.2011.05005.x#support-information-section |

Table S5.

| **Name of strain** | **Lab ID** | **Origin** | **Genotype** | **Note** |
| --- | --- | --- | --- | --- |
| WN | OS720 | This study | hoΔ::HphMX MATa/alpha ura3-delta0 leu2-delta0 lys2-delta0 | WE × NA |
| WE(*GAT1*) | OS721 | This study | *GAT1*::KanMX hoΔ::HphMX MATa ura3-delta0 leu2-delta0 lys2-delta0 |  |
| NA(*GAT1*) | OS722 | This study | *GAT1*::KanMX hoΔ::HphMX MATalpha ura3-delta0 leu2-delta0 lys2-delta0 |  |
| Δ/NA(*GAT1*) | OS723 | This study | (OS721×OS605) MATa/alpha *GAT1*::KanMX/NA*GAT1*  hoΔ::HphMX/hoΔ::HphMX ura3/ura3 leu2/leu2 lys2/lys2 | *GAT1*（NA）hemizygote |
| WE(*GAT1*)/Δ | OS724 | This study | (OS587×OS722) MATa/alpha WE*GAT1*/*GAT1*::KanMX  hoΔ::HphMX/hoΔ::HphMX ura3/ura3 leu2/leu2 lys2/lys2 | *GAT1*（WE）hemizygote |
| WE(*BST1*) | OS725 | This study | *BST1*::KanMX hoΔ::HphMX MATa ura3-delta0 leu2-delta0 lys2-delta0 |  |
| NA(*BST1*) | OS726 | This study | *BST1*::KanMX hoΔ::HphMX MATalpha ura3-delta0 leu2-delta0 lys2-delta0 |  |
| Δ/NA(*BST1*) | OS727 | This study | (OS725×OS605) MATa/alpha *BST1*::KanMX/NA*BST1*  hoΔ::HphMX/hoΔ::HphMX ura3/ura3 leu2/leu2 lys2/lys2 | *BST1*（NA）hemizygote |
| WE(*BST1*)/Δ | OS728 | This study | (OS587×OS726) MATa/alpha WE*BST1*/*BST1*::KanMX  hoΔ::HphMX/hoΔ::HphMX ura3/ura3 leu2/leu2 lys2/lys2 | *BST1*（WE）hemizygote |
| WE(*SAT4*) | OS741 | This study | *SAT4*::KanMX hoΔ::HphMX MATa ura3-delta0 leu2-delta0 lys2-delta0 |  |
| NA(*SAT4*) | OS742 | This study | *SAT4*::KanMX hoΔ::HphMX MATalpha ura3-delta0 leu2-delta0 lys2-delta0 |  |
| Δ/NA(*SAT4*) | OS743 | This study | (OS741×OS605) MATa/alpha *SAT4*::KanMX/NA*SAT4*  hoΔ::HphMX/hoΔ::HphMX ura3/ura3 leu2/leu2 lys2/lys2 | *SAT4*（NA）hemizygote |
| WE(*SAT4*)/Δ | OS744 | This study | (OS587×OS742) MATa/alpha WE*SAT4*/*SAT4*::KanMX  hoΔ::HphMX/hoΔ::HphMX ura3/ura3 leu2/leu2 lys2/lys2 | *SAT4*（WE）hemizygote |
| WE(*ADY2*) | OS745 | This study | *ADY2*::KanMX hoΔ::HphMX MATa ura3-delta0 leu2-delta0 lys2-delta0 |  |
| NA(*ADY2*) | OS746 | This study | *ADY2*::KanMX hoΔ::HphMX MATalpha ura3-delta0 leu2-delta0 lys2-delta0 |  |
| Δ/NA(*ADY2*) | OS747 | This study | (OS745×OS605) MATa/alpha *ADY2*::KanMX/NA*ADY2*  hoΔ::HphMX/hoΔ::HphMX ura3/ura3 leu2/leu2 lys2/lys2 | *ADY2*（NA）hemizygote |
| WE(*ADY2*)/Δ | OS748 | This study | (OS587×OS746) MATa/alpha WE*ADY2*/*ADY2*::KanMX  hoΔ::HphMX/hoΔ::HphMX ura3/ura3 leu2/leu2 lys2/lys2 | *ADY2*（WE）hemizygote |
| WE(*ARI1*) | OS749 | This study | *ARI1*::KanMX hoΔ::HphMX MATa ura3-delta0 leu2-delta0 lys2-delta0 |  |
| NA(*ARI1*) | OS750 | This study | *ARI1*::KanMX hoΔ::HphMX MATalpha ura3-delta0 leu2-delta0 lys2-delta0 |  |
| Δ/NA(*ARI1*) | OS751 | This study | (OS749×OS605) MATa/alpha *ARI1*::KanMX/NA*ARI1*  hoΔ::HphMX/hoΔ::HphMX ura3/ura3 leu2/leu2 lys2/lys2 | *ARI1*（NA）hemizygote |
| WE(*ARI1*)/Δ | OS752 | This study | (OS587×OS750) MATa/alpha WE*ARI1*/*ARI1*::KanMX  hoΔ::HphMX/hoΔ::HphMX ura3/ura3 leu2/leu2 lys2/lys2 | *ARI1*（WE）hemizygote |
| WE(*YFL040W*) | OS729 | This study | *YFL040W*::KanMX hoΔ::HphMX MATa ura3-delta0 leu2-delta0 lys2-delta0 |  |
| NA(*YFL040W*) | OS730 | This study | *YFL040W*::KanMX hoΔ::HphMX MATalpha ura3-delta0 leu2-delta0 lys2-delta0 |  |
| Δ/NA(*YFL040W*) | OS731 | This study | (OS729×OS605) MATa/alpha *YFL040W*::KanMX/NA*YFL040W*  hoΔ::HphMX/hoΔ::HphMX ura3/ura3 leu2/leu2 lys2/lys2 | *YFL040W*（NA）hemizygote |
| WE(*YFL040W*)/Δ | OS732 | This study | (OS587×OS730) MATa/alpha WE*YFL040W*/*YFL040W*::KanMX  hoΔ::HphMX/hoΔ::HphMX ura3/ura3 leu2/leu2 lys2/lys2 | *YFL040W*（WE）hemizygote |
| WE(*FUB1*) | OS733 | This study | *FUB1*::KanMX hoΔ::HphMX MATa ura3-delta0 leu2-delta0 lys2-delta0 |  |
| NA(*FUB1*) | OS734 | This study | *FUB1*::KanMX hoΔ::HphMX MATalpha ura3-delta0 leu2-delta0 lys2-delta0 |  |
| Δ/NA(*FUB1*) | OS735 | This study | (OS733×OS605) MATa/alpha *FUB1*::KanMX/NA*FUB1*  hoΔ::HphMX/hoΔ::HphMX ura3/ura3 leu2/leu2 lys2/lys2 | *FUB1*（NA）hemizygote |
| WE(*FUB1*)/Δ | OS736 | This study | (OS587×OS734) MATa/alpha WE*FUB1*/*FUB1*::KanMX  hoΔ::HphMX/hoΔ::HphMX ura3/ura3 leu2/leu2 lys2/lys2 | *FUB1*（WE）hemizygote |
| WE(*PTC6*) | OS737 | This study | *PTC6*::KanMX hoΔ::HphMX MATa ura3-delta0 leu2-delta0 lys2-delta0 |  |
| NA(*PTC6*) | OS738 | This study | *PTC6*::KanMX hoΔ::HphMX MATalpha ura3-delta0 leu2-delta0 lys2-delta0 |  |
| Δ/NA(*PTC6*) | OS739 | This study | (OS737×OS605) MATa/alpha *PTC6*::KanMX/NA*PTC6*  hoΔ::HphMX/hoΔ::HphMX ura3/ura3 leu2/leu2 lys2/lys2 | *PTC6*（NA）hemizygote |
| WE(*PTC6*)/Δ | OS740 | This study | (OS587×OS738) MATa/alpha WE*PTC6*/*PTC6*::KanMX  hoΔ::HphMX/hoΔ::HphMX ura3/ura3 leu2/leu2 lys2/lys2 | *PTC6*（WE）hemizygote |

Table S6.

| PCR for transformation |  |  |
| --- | --- | --- |
| Step | Time and Temperature | Note |
| Hot start | 95°C, 5 min |  |
| Denaturation | 95°C 30 sec | 35 cycles |
| Annealing | 55°C 30 sec |  |
| Extension | 72°C 3 min/kb |  |
| Final extension | 72°C 10 min |  |
| PCR for validation |  |  |
| Hot start | 95°C, 5 min |  |
| Denaturation | 95°C 30 sec | 35 cycles |
| Annealing | 56°C 30 sec |  |
| Extension | 72°C 1 min |  |
| Final extension | 72°C 5 min |  |
| PCR for mating type |  |  |
| Hot start | 95°C, 5 min |  |
| Denaturation | 95°C 30 sec | 35 cycles |
| Annealing | 55°C 30 sec |  |
| Extension | 72°C 2 min |  |
| Final extension | 72°C 10 min |  |
